# Pain in monogenic Parkinson’s disease: a comprehensive review

**DOI:** 10.3389/fneur.2023.1248828

**Published:** 2023-10-30

**Authors:** Parisa Alizadeh, Cinthia Terroba-Chambi, Beatrice Achen, Veronica Bruno

**Affiliations:** ^1^Department of Clinical Neurosciences, University of Calgary, Calgary, AB, Canada; ^2^Hotchkiss Brain Institute, Calgary, AB, Canada

**Keywords:** Parkinson’s disease, monogenic, genetic, pain, inheritance

## Abstract

Pain, a challenging symptom experienced by individuals diagnosed with Parkinson’s disease (PD), still lacks a comprehensive understanding of its underlying pathophysiological mechanisms. A systematic investigation of its prevalence and impact on the quality of life in patients affected by monogenic forms of PD has yet to be undertaken. This comprehensive review aims to provide an overview of the association between pain and monogenic forms of PD, specifically focusing on pathogenic variants in *SNCA*, *PRKN*, *PINK1*, *PARK7*, *LRRK2*, *GBA1, VPS35, ATP13A2, DNAJC6, FBXO7*, and *SYNJ1*. Sixty-three articles discussing pain associated with monogenic PD were identified and analyzed. The included studies exhibited significant heterogeneity in design, sample size, and pain outcome measures. Nonetheless, the findings of this review suggest that patients with monogenic PD may experience specific types of pain depending on the pathogenic variant present, distinguishing them from non-carriers. For instance, individuals with *SNCA* pathogenic variants have reported painful dystonia, lower extremity pain, dorsal pain, and upper back pain. However, these observations are primarily based on case reports with unclear prevalence. Painful lower limb dystonia and lower back pain are prominent symptoms in *PRKN* carriers. A continual correlation has been noted between *LRRK2* mutations and the emergence of pain, though the conflicting research outcomes pose challenges in reaching definitive conclusions. Individuals with *PINK1* mutation carriers also frequently report experiencing pain. Pain has been frequently reported as an initial symptom and the most troublesome one in *GBA1*-PD patients compared to those with idiopathic PD. The evidence regarding pain in *ATP13A2, PARK7, VPS35, DNAJC6, FBXO7,* and *SYNJ1*pathogenic variants is limited and insufficient. The potential linkage between genetic profiles and pain outcomes holds promising clinical implications, allowing for the potential stratification of patients in clinical trials and the development of personalized treatments for pain in monogenic PD. In conclusion, this review underscores the need for further research to unravel the intricate relationship between pain and monogenic forms of PD. Standardized methodologies, larger sample sizes, and longitudinal studies are essential to elucidate the underlying mechanisms and develop targeted therapeutic interventions for pain management in individuals with monogenic PD.

## 1. Introduction

Parkinson’s disease (PD) is a complex disorder with significant clinical variability, potentially influenced by genetic factors, affecting not only motor but also non-motor symptoms (NMS) including pain (1, 2). Pain in PD encompasses various categories, including musculoskeletal pain, chronic pain, fluctuation-related pain, nocturnal pain, orofacial pain, discoloration, edema/swelling, and radicular pain, as categorized by the King’s Parkinson’s Disease Pain Scale (3).

Despite of its high prevalence, with reports of up to 85% of PD patients experiencing pain (4–6), it remains underdiagnosed and undertreated, even if it significantly impacts the quality of life (7–12). Lower Back Pain (LBP) is the most common pain site in PD, surpassing its prevalence in healthy older adults (13). Shoulder pain can even precede the PD diagnosis (14), with 12% of PD patients reporting it as their initial symptom (15). Motor complications in PD patients correlate with a higher risk of pain, and pain potentially exacerbates parkinsonian symptoms (5, 16). Given the complexity of pain etiology and the limited therapeutic options for its management, a comprehensive and accurate classification of pain types are crucial for improved patient outcomes (12).

Pathogenic variants in PD-causative genes have been associated with diverse disease symptoms (17, 18). For example, cognitive decline affects 70% of PD patients with pathogenic alpha-synuclein (*SNCA*) gene variants, while only 23% of Leucine-Rich Repeat Kinase 2 (*LRRK2*) carriers exhibit cognitive impairment (18). Rigidity and bradykinesia are nearly universal in *SNCA* patients, with dystonia less frequently observed (18). Despite these observations, data on pain in monogenic forms of PD remain limited. Case reports and small case–control studies indicate variations in pain presentation among different genetic forms of PD. For instance, a PD patient with an *SNCA* pathogenic variant exhibited dorsal pain as a primary symptom (19), while another patient with the Leu347Pro PTEN-induced putative protein kinase 1 (*PINK1*) pathogenic variant developed long-term right-sided pain following right-hand tremor onset (20).

Reports also suggest pain as an initial symptom in PD patients with the G2019S *LRRK2* variant (21). Yet, these reports rarely explore the longitudinal progression of pain or compare pain experiences among carriers of different pathogenic variants under similar conditions. Consequently, the question of whether genetic status directly leads to the emergence of pain or is associated with different types in PD remains unanswered.

This review aims to investigate the hypothesis that genotypes may influence the pain phenotype in PD patients. We assess the presence, types, severity, and onset time of pain in PD patients and their relationship with different variants in pathogenic genes. By synthetizing existing literature, this review seeks to enhance our understanding of pain in monogenic forms of PD and offer insights for future research and clinical management.

## 2. Methods

### 2.1. Search strategy

To ensure methodological rigor, this review adhered to the Preferred Reporting Items for Systematic Reviews and Meta-Analyses extension for Scoping Reviews (PRISMA-ScR) (22). The initial literature search encompassed databases such as MEDLINE (PubMed), Embase (Elsevier), and Cochrane databases. We sought articles published from inception until March 2023. The search strategy employed a combination of subject headings and MeSH terms, including “Parkinson’s disease” OR “Parkinson disease” OR “PD,” AND “gene” OR “genetic” OR “monogenic” OR “*SNCA*” OR “PRKN” OR “Parkin” OR “PINK1” OR “DJ1” OR “LRRK2” OR “ATP13A2” OR “GBA1” OR “DNAJC6” OR “FBXO7” OR “SYNJ1” OR “PARK1” OR “PARK2” OR “PARK4” OR “PARK6” OR “PARK7” OR “PARK8” OR “PARK9” OR “PARK15” OR “PARK19” OR “PARK20,” AND “pain” OR “pain sensation” OR “somatosensory discomfort.” The search was conducted without restrictions on language, year of publication, study type, or publication status.

Two independent investigators (PA and CTC) conducted the search, and the search results, including abstracts and full-text articles, were organized using reference management software. In addition to the electronic search, references from included studies and review articles were screened to augment the dataset.

Monogenic forms of PD result from the inheritance of a pathogenic variant of a single gene, contributing to approximately 30% of familial cases and 3–5% of sporadic cases (23). While the PD causative gene landscape has sparked some debate, several genes, including, *SNCA*, Parkin RBR E3 Ubiquitin Protein Ligase (*PRKN*), *PINK1*, *LRRK2*, and deglycase DJ-1 (*PARK7*) are widely as acknowledged as monogenic PD genes by most experts (24, 25). This review incorporates vacuolar protein sorting 35 (*VPS35*), with only one confirmed pathogenic variant (26), ATPase Cation Transporting 13A2 (*ATP13A2*), F- box protein 7 (*FBXO7*), DnaJ Heat Shock Protein Family (Hsp40) Member C6 (*DNAJC6*), and Synaptojanin-1 (*SYNJ1*) based on a recent comprehensive review (25).

We have included findings related to the *GBA1* gene (Glucosylceramidase) which elevate the risk of developing PD. Pain reports are common among *GBA1* carriers, making this addition significant to our review (27).

Specific pathogenic variants linked to PD and pain syndromes are discussed within the text, while those variants reported only in individual cases are further summarized in Table 1 for comprehensive reference.

**Table 1:** Pain in monogenic PD: summary of the data extracted from the included studies concerning clinical studies.

Gene/ Symbol/ Inheritance	Protein product	Variants type	Authors/year	Type of study	Sample size	Pain-related information	Other clinical features
*SNCA*/ PARK1, PARK4/ AD	Alpha-synuclein	*SNCA* missense (H50Q)	Appel-Cresswell et al. (2013) (28)	Case series/report	110 fully sequenced 1105 patients and 875 control TaqMan sequencing	Painful dystonic flexion on walking in carriers.	Bilateral action tremor, micrographia, and decreased walking speed with shuffling.
		*SNCA* missense (G51D)	Lesage et al. (2013) (29)	Case series/report	4 patients	Lower extremity pain in 1 out of 3 carriers.	Left hemibody rest tremor.
		Mosaicism of duplication and triplication in oral mucosal cells	Perandones et al. (2014) (19)	Case series/report	2 cases	Dorsal pain in 1 out of 2 patients	First presented with dorsal pain and gait disorders, secondary to rigidity and bradykinesia of the lower left leg.
		Triplication	Byers et al. (2011) (30)	Case series/report	1 case	Upper back pain	Fatigue, tremors, and decreased dexterity as initial symptoms.
*PRKN*/ PARK2/ AR	Parkin	Homozygous Exon 3 deletion	Capecci et al. (2004) (31)	Case series/report	1 patient	Painful dystonic posture during off phases.	Psychomotor slowness, mood depression, insomnia.
		Intron 5 splice mutation/intron 5 splice mutation and exon 8 deletion	Khan et al. (2002) (32)	Case series/report	10 patients	Lower limb pain/leg pain, pain with “OFF-periods” and painful dystonic cramps of the feet.	Bilateral leg tremor.
		Homozygous exon 4 deletion	Dogu et al. (2004) (33)	Case series/report	12 siblings	Left foot severe pain as first complaint and left foot dystonia two years later in one patient.	N/A
		Compound heterozygous Parkin mutation (a deletion of exon 7 and a missense mutation in exon 12)	Djarmati et al. (2004) (34)	Case series/report	75 unrelated patients	4% with pain and 24% with dystonia as their onset symptom. No clarification about Parkin mutation carriers was among them.	N/A
		Different deletions*	Ohsawa et al. (2005) (35)	Case series/report	9 Parkin patients and 8 idiopathic PD	Tingling sensation with foot sensory loss in 2 out of 9 Parkin patients. Significant decrease of SNAP amplitude in 8 out of 9 Parkin patients.	N/A
		1 homozygous exon 2 deletion, 13 compound heterozygous, and 10 had single mutant allele	Khan et al. (2003) (36)	Case series/report	115 PD patients (24 Parkin patients)	Painful ‘OFF’ periods in homozygous patients.	N/A
		Homozygous for 202A deletion	Nisipeanu et al. (2001) (37)	Case series/report	4 brothers	Low back pain	N/A
		3 *PRKN* deletion patients (2 homozygous and 1 heterozygous)	Bouhouche et al. (2017) (38)	Case series/report	18 consanguineous patients	No pain for 3 Parkin patients.	N/A
		9 homozygous deletion mutations and 7 had a heterozygous point mutation	Shyu et al. (2005) (39)	Cross-sectional	230 PD (30 Parkin carriers)	50% of patients with tingling pains over both lower legs.	The same patients complained of profound dizziness.
		15 heterozygous, 3 homozygous, and 7 compound heterozygous including different kinds of deletions or duplications	Monroy-Jaramillo et al. (2014) (40)	Cross-sectional	122 non-related EOPD patients (25 Parkin mutation) and 120 HC	The patient with exon 9 deletion experienced pain.	N/A
		93 carried two mutations and 25 had one mutation	Lesage et al. (2007) (41)	Cross-sectional	435 patients	Painful contractions with objective mild sensory neuropathy in the lower limbs 1 out of 3 sisters with single heterozygous deletion of exon 3.	Left foot dystonia in the other sisters. No pain was mentioned. Another sister had a mild decrease in sensory nerve action potentials in the lower limbs without pain.
		Different Parkin mutations	Doherty et al. (2013) (42)	Case-control	5 Parkin, 5 pathologically confirmed PD, and 4 HC	FOG and painful OFF-period dystonia in *PRKN* carriers.	N/A
		Parkin heterozygous mutation	Gierthmühlen et al. (2010) (43)	Case-control	9 Parkin carriers and 9 HC	Somatosensory disturbances (Sensory gain for the cold pain threshold in 5 out 9 parkin carriers. Sensory gain for the hot pain threshold in one carrier).	N/A
		Different kinds of mutation	Koziorowski et al. (2013) (44)	Case-control	150 EOPD patients and 230 HC	43% of Parkin carriers had “other symptoms” including pain and dystonia as an onset symptom in comparison with 13 % for non-carriers.	N/A
		202A deletion (12 homozygous, 1 heterozygous)	Hassin-Baer et al. (2011) (45)	Cohort	13 PD patients and 15 family members	Severe LBP (8 out of 13) Painful dystonia (2 out of 13)	N/A
		Different Parkin mutations	Elia et al. (2014) (46)	Cohort	44 patients	Lower limb pain in 3 patients.	All patients had lower limb walking task-specific dystonia.
*PINK1*/ PARK6/ AR	PTEN-induced putative kinase 1	One with homozygous transition in exon 7 (Q456>X)	Zadikoff et al. (2006) (47)	Case series/report	11 PD patients	Back, and extremity pain, and painful wearing-off dystonia are frequent complaints.	N/A
		Homozygous A217D mutation	Norman et al. (2017) (48)	Case series/report	1 EOPD patient (family of Moroccan origin)	Back and shoulder pain.	N/A
		5 heterozygous for Arg246Gln & Arg276Gln	Biswas et al. (2010) (49)	Case series/report	250 patients and 205 HC	Pain in legs, calves, knees, spine, and back.	N/A
		27 variants including 1 homozygous T→C substitution in exon 5 (Leu347Pro)	Rogaeva et al. (2004) (20)	Case series/report	289 PD patients and 80 HC in the first stage and 150 HC for estimating the mutation frequencies	Pain on the right side after 10 years.	Right-hand tremor as an onset symptom.
		1 homozygous L347P	Kilarski et al. (2012) (50)	Case series/report	136 EOPD	Pain as an onset symptom.	Lower limb tremor as an onset symptom.
		1 homozygous nonsense mutation in exon 3 (Tyr258Stop)	Tan et al. (2006) (51)	Case series/report	80 sporadic EOPD patients	Painful paresthesia	An urge to move her lower limbs was accompanied by painful paresthesia with a cramp-like feeling distally.
		1 homozygous L539F *PINK1* and 1 homozygous Q456X *PINK1* mutation	Bouhouche et al. (2017) (52)	Case series/report	19 unrelated PD patients	No pain reported	N/A
		Different mutations in exon2*	Djarmati et al. (2006) (53)	Cross-sectional	92 EOPD patients	Right shoulder pain in 2 heterozygous patients [mutation (952A>T in exon 2: Met318Leu)]	Four variants were found and three of them (c.558GC, c.626CT, and c.952AT) are likely to be pathogenic.
		Different kinds of mutations	Ibanez et al. (2006) (54)	Case-control	53 patients without *PINK1* mutations 34 *PINK1* patients, and 174 HC	Painful episodes of torticollis and levodopa-induced painful dystonia episodes in 1 homozygous Q456X *PINK1* mutation patient.	N/A
		*PINK1* mutation	Gierthmühlen et al. (2009) (55)	Case-control	14 family members with *PINK1* mutation and 14 HC	Somatosensory impairment (higher mechanical pain, and pain pressure thresholds in *PINK1* carriers than HC).	N/A
		Different substitution mutations*	Koziorowski et al. (2013) (44)	Case-control	150 EOPD patients and 230 HC	No pain reported	N/A
*LRRK2*/ PARK8/ AD	Leucine-rich repeat kinase 2	Heterozygous N1437H mutation	Puschmann et al. (2012) (56)	Case series/report	1 patient for clinical study and 7 brains for genetic study.	Severe painful dystonia in ON state.	N/A
		11 G2019S mutation	Bras et al. (2005) (57)	Case series/report	128 PD patients	Painful cervical dystonia was responsive to levodopa in 2 carriers.	N/A
		3 G2019S mutation (2 familial and 1 idiopathic patient)	Gosal et al. (2005) (58)	Case series/report	273 PD patients	Idiopathic patient Painful left foot dystonia after 9 years of onset symptoms in an idiopathic patient.	The same patient had heaviness in the right arm and leg, which caused some walking difficulties as an onset symptom.
		One p.R1441G mutation, one p.G2019S, and 103 G2385R	Hatano et al. (2014) (59)	Case series/report	871 PD patients (430 sporadic PD and 441 probands with familial PD)	Severe wasting painful dyskinesia after 13 years of disease onset in a patient with both R1441G and G2385R mutations in *LRRK2*.	Bradykinesia and tremors in the left lower limb as onset symptoms.
		Three with G2019S mutation	Gatto et al. (2013) (21)	Case series/report	55 PD patients	Pain as an onset symptom in one carrier with abnormal MMSE.	N/A
		*LRRK2* mutation	Khlebtovsky et al. (2018) (60)	Cross-sectional	28 PD patients	Higher heat pain threshold in LRRK2 carriers than non-carriers.	N/A
		2 with R1441C and 2 with G2019S mutation	Hedrich et al. (2006) (61)	Cross-sectional	First included: 98 EOPD, 42 LOPD patients. Further included: 220 EOPD patients and 200 HC	Joint pain was the initial symptom in one patient with R1441C LRRK2 mutation.	N/A
		8 heterozygous R1441C mutation, 1 heterozygous G2019S mutation	Criscuolo et al. (2011) (62)	Cross-sectional	192 PD patients	Pain in 5 R1441C carriers vs. one no-carrier	N/A
		G2019S mutation	Bouhouche et al. (2017) (52)	Cross-sectional	100 unrelated PD patients	No significant difference in pain prevalence.	N/A
		G2385R or R1628P LRRK2 variants	Li et al. (2015) (63)	Cross-sectional	1225 PD patients	No differences in the NMS phenotype.	N/A
		*LRRK2* Gly2019Ser mutation	Healy et al. (2008) (64)	Case-control	24 world populations, 19 376 patients	126 out of 301 *LRRK2* PD patients (42%) had dystonia, mostly painful foot dystonia "OFF-period"(25%for idiopathic PD).	N/A
		7 PD patients and 2 PD relatives (at-risk group) had *LRRK2* mutation	Baig et al. (2015) (65)	Case-control	769 PD patients, 98 at risk (first-degree PD relatives), and 287 HC	The pain was reported among symptoms in 55.6% of PD patients and 1.2% of relatives.	N/A
		G2385R mutation	An et al. (2008) (66)	Case-control	600 PD patients and 334 unrelated HC	No significant difference between genotypes in pain as an onset symptom.	N/A
		*LRRK2* G2385R or R1628P	Wang et al. (2014) (67)	Case-control	223 *LRRK2*-PD carriers and 1366 iPD.	No difference in pain between *LRRK2* PD patients and idiopathic PD.	N/A
		*LRRK2* R1628P mutation	Zhang et al. (2009) (68)	Case-control	600 patients and 459 unrelated HC	No significant difference in pain as an onset symptom among genotypes.	N/A
		7 heterozygous G2019S mutation	Luciano et al. (2010) (69)	Cohort	791 individuals	Knee pain was reported among symptoms in one individual who developed PD.	N/A
*ATP13A2*/ PARK9/ AR	ATPase 13A2	1 homozygous deletion (c.2822delG)	Martino et al. (2015) (70)	Case series/report	1 PD patient	Pain in the right hand	Right-arm dystonic posturing is an onset symptom.
		Two with W258X mutation	Bouhouche et al. (2017) (52)	Cross-sectional	19 PD patients	No pain among their symptoms.	N/A
DJ-1/ *PARK7*/ AR	DJ-1	1 heterozygous deletion of exon5	Djarmati et al. (2004) (34)	Case series/report	75 unrelated PD patients	Pain as an onset symptom in 3 out of 75 (4%). No mention if the DJ-1 carrier was one of them.	N/A
*GBA1*/ AD	Glucocerebrosidase	15 with *GBA* mutations	Bonner et al. (2020) (71)	Case series/report	20 PD patients (15 with PD-*GBA* and 5 with idiopathic PD)	The pain was reported as the most bothersome symptom in 17 patients (12 *GBA*-PD patients) *GBA*-PD patients reported rigidity and stiffness often combined with pain.	Sleep disruption was reported as caused by pain in 2 patients. No clarification if patients were *GBA* carriers.
		N370S (homozygous)	Rodriguez-Porcel et al. (2017) (72)	Case series/report	2 *GBA*-PD patients	A cramp-like pain	N/A
		3 heterozygous D409H and 1 heterozygous R463H	Kresojevic et al. (2015) (73)	Cross-sectional	578 PD patients	Pain is an initial symptom in all carrier patients.	N/A
		Different heterozygous mutations	Jesús et al. (2016) (74)	Case-control	532 iPD patients (62 carriers) and 542 HC (43 carriers)	37.9% of deleterious and 40% of benign *GBA* carriers vs. 34.4% of non-carriers had pain	Among other NMS, REM sleep disorder was significantly more common among *GBA* carriers than non-carriers
		12 heterozygous including five N370S, two L444P, and other different mutations	McNeill et al. (2012) (75)	Case-control	220 PD patients (12 PD-*GBA* and 20 non-*GBA* mutations PD patients)	Unexplained pain was more common among *GBA*-PD patients than sporadic (58% vs. 10%, p=0.005).	N/A
		L444P mutation	Wang et al. (2014) (67)	Case-control	49 *GBA*-PD and 1366 iPD	No differences concerning bodily pain between groups.	N/A
		Different kinds of point mutations or deletion	Neumann et al. (2009) (76)	Case-control	790 PD and 257 controls	A patient with R463C mutation experienced pain in the left shoulder and lower back pain, also a patient with G193E reported back pain	N/A

*Type of mutations is fully described in the manuscript.

*SNCA*, alpha-synuclein; *PRKN*, Parkin RBR E3 Ubiquitin Protein Ligase; *PINK1*, PTEN induced putative protein kinase 1; *LRRK2*, Leucine-Rich Repeat Kinase 2; *ATP13A2*, ATPase Cation Transporting 13A2; DJ-1, deglycase; and *GBA*, Glucosylceramidase; N/A, non-applicable; PD, Parkinson’s disease; iPD, idiopathic Parkinson’s disease; HC, healthy controls; AA, AG, GG, different genotypes of SNPs which have different base pair; FOG, Freezing of gait; EOPD, Early-onset Parkinson’s disease; SNAP, Sural Sensory Nerve Action Potential; T→C, T to C transition (mutation); MMSE, Mini-Mental State Examination; PD-*GBA*, *GBA* carrier PD patients.

### 2.2. Selection criteria

To ensure the relevance and quality of selected articles, the following inclusion criteria were applied: (A) Published papers that specifically focused on pain symptoms in patients with monogenic pathogenic variants associated with PD and (B) Articles that provided information on autosomal dominant (AD) or recessive (AR) forms of PD or on patients carrying at least one pathogenic variant in the *SNCA*, *PRKN*, *PINK1*, *PARK7*, *LRRK2*, *GBA1*, *VPS35*, *ATP13A2*, *DNAJC6*, *FBXO7*, or *SYNJ1* genes, and reporting cases of PD-related pain. Furthermore, a meta-summary was conducted to consolidate the overall findings from the selected studies.

The exclusion criteria were as follows: (A) Articles that included PD patients carrying gene pathogenic variants other than those listed above or patients with other pain-related diseases, X-linked dystonia-parkinsonism, or rapid-onset dystonia-parkinsonism, and (B) Redundant publications.

The assessment of the retrieved occurred in two phases. Initially, titles and abstracts were screened based on the inclusion/exclusion criteria. Subsequently, the full text of the remaining articles was reviewed for final selection. Any articles that did not provide pertinent information regarding pain in monogenic forms of PD, even after a thorough full-text revision, were excluded from the analysis.

### 2.3. Specific aim

This review aimed to investigate the presence of pain in individuals with monogenic variants associated with PD. The primary objectives were to ascertain whether particular gene pathogenic variants within the spectrum of monogenic PD genes correlate with the presence of pain and, more specifically, to explore whether these pathogenic variants are associated with specific types of pain.

## 3. Results

### 3.1. Identification of studies

A consolidated master list comprising 541 potentially eligible articles was generated from the contributions of the two reviewers. Duplicate entries within the list were identified and removed, resulting in 534 unique articles. A preliminary screening of the titles and abstracts was conducted by the study team, which led to the exclusion of 3 due to the lack of relevance to the search terms. Furthermore, several papers were flagged for full-text review but were subsequently excluded as they did not contain any mention of pain within the reported symptoms. This process led to the exclusion of 463 articles.

**Figure 1:**
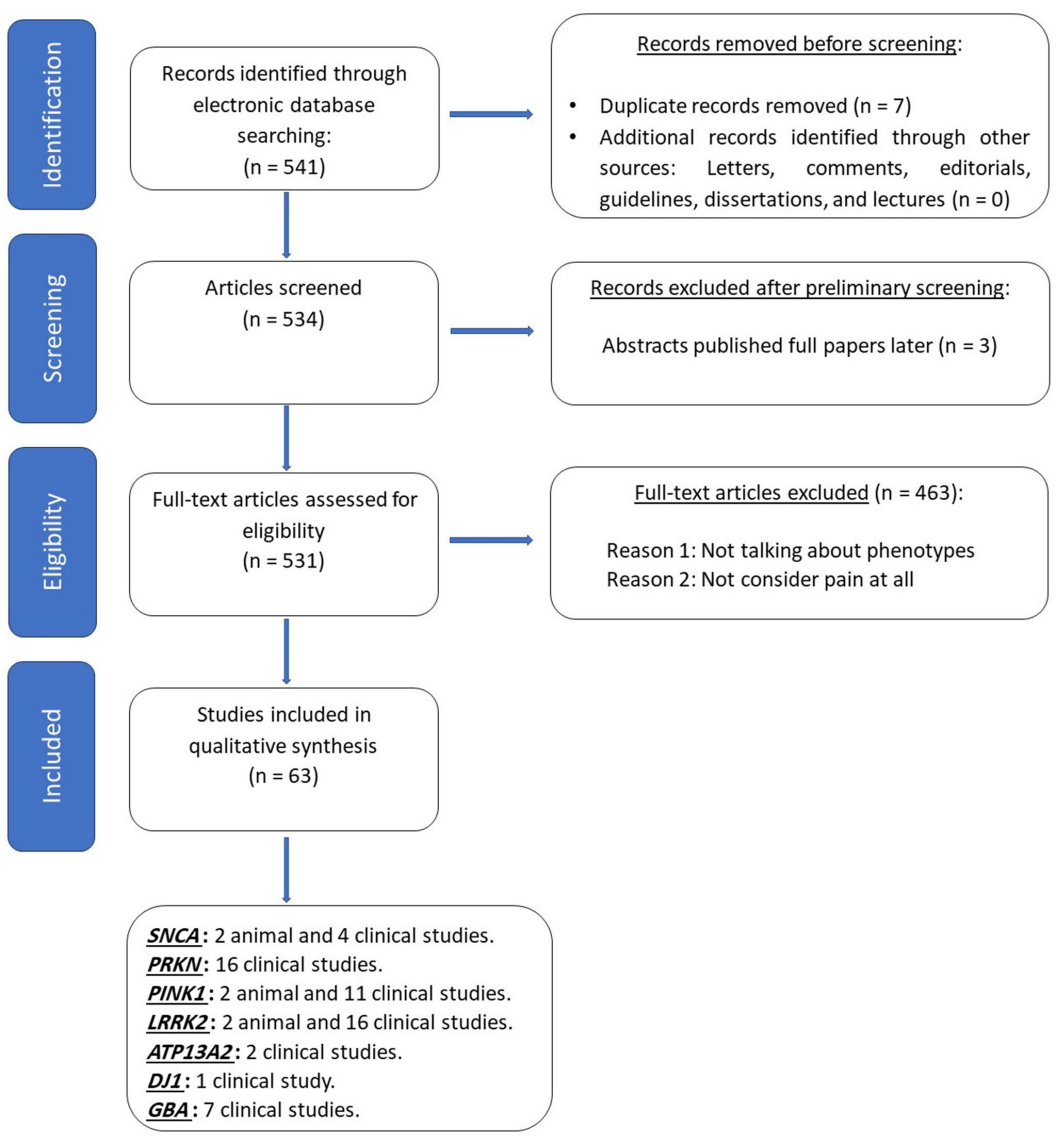
PRISMA’s diagram depicts the flow of information through the different phases of the comprehensive review, which included searches of databases. *SNCA*, alpha- synuclein; *PRKN*, Parkin RBR E3 Ubiquitin Protein Ligase; *PINK1*, PTEN induced putative protein kinase 1; LRRK2, Leucine-Rich Repeat Kinase 2; ATP13A2, ATPase Cation Transporting 13A2; *PARK7*, deglycase; *GBA*, Glucosylceramidase.

In total, 63 articles met eligibility criteria for inclusion in this review. These selected articles encompassed six studies conducted on animal models, twenty-eight case series or case reports, eleven cross-sectional studies, fifteen case–control studies, and three prospective cohort studies (Figure 1). Each selected study underwent a comprehensive review, and pertinent data were extracted.

For each gene, we provide a general description and a summary of clinical studies, while details related to animal models will be presented in Table 2.

### 3.2. Monogenic forms of PD

More than 40 distinct chromosomal loci and 21 disease-causing genes associated with PD have been identified (77, 78). Among these, specific regions house known genes responsible for monogenic PD. Recognized monogenic PD genes include *SNCA, PRKN, PINK1, PARK7, LRRK2, GBA1, VPS35, ATP13A2, DNAJC6, FBXO7,* and *SYNJ1* (25, 78). In monogenic PD, a pathogenic variant in a single gene is sufficient to manifest the PD phenotype (79).

All of the above mentioned genes exhibit autosomal inheritance patterns (79). In general, phenotypes resembling idiopathic PD (iPD) are more commonly observed in cases of AD inheritance, whereas young-onset parkinsonism resembling iPD or parkinsonism with atypical features is more commonly associated with AR inheritance (78).

Our review encompasses 11 out of 19 known PD-causing genes identified in the most recent comprehensive genetic database of PD (80). The exclusion of the remaining genes is due to insufficient data supporting their pathogenic role in PD and subsequent studies failed to replicate the pathogenic variant (81, 82).

Approximately 15% of individuals with PD have a family history of the disorder, with the current known monogenic forms accounting for approximately 30% of familial PD cases (16, 83).

The selected articles included in this review primarily focus on genes *SNCA, PRKN, LRRK2, PINK1, PARK7,* and *GBA1* which are associated with either Early Onset PD (EOPD) or Late Onset PD (LOPD). We also sought information on *ATP13A2, DNAJC6, FBXO7,* and *SYNJ1,* which are rare causes of atypical PD However, our search did not yield any reports specifically addressing pain in relation to these genes.

A summary of the review results is provided in Table 1, providing an overview of key findings related to of pain in the context of monogenic PD.

**Table 2:** Pain in monogenic PD: summary of the data extracted from the included studies concerning animal models.

Gene/ Symbol/ Inheritance	Protein product	Variants type	Authors/year	Sample size	Pain-related information
*SNCA*/ PARK1, PARK4/ AD	Alpha-synuclein	Higher expression of alpha-Synuclein	Vivacqua et al. (2009) (84)	At least 3 rats	The abnormal pain in PD may be caused by the pathological changes related to alpha-Synuclein
		*SNCA* missense (A53T)	Valek et al. (2021) (85)	32 mice	*PINK1*-/-*SNCA* A53T double mutant mice show early prodromal sensory neuropathy. Loss of thermal sensitivity is an initial sign of sensory dysfunction.
*PINK1*/ PARK6/ AR	PTEN-induced putative kinase 1	*Pink1*–/–	Johnson et al. (2020) (86)	Rat model in PD	Abnormal nociceptive responses and faster thermal withdrawal latencies in *PINK1* -/- rats.
		*Pink1*–/–	Yi et al. (2019) (87)	Rat model in PD	*PINK1*-positive cells participate in the development of pain following mitochondrial autophagy.
*LRRK2*/ PARK8/ AD	Leucine-rich repeat kinase 2	R1441G mutation in *LRRK2*	Bichler et al. (2013) (88)	*LRRK2* BAC (Bacterial Artificial Chromosome) transgenic (Tg) mice and control ones (NTg)	Pain sensitivity.
		Gain of function mutation in *LRRK2*	Valek et al. (2019) (89)	*LRRK2*/Park8 transgenic PD mice and rats	Not develop any sensory deficits.

No animal studies concerning pain in the following gene mutations were found: *PRKN*/PARK2 (Parkin), *ATP13A1*/PARK9 (ATPase 13A2), DJ-1/*PARK7* (deglycase), *GBA1* (Glucocerebrosidase).

*SNCA*, alpha-synuclein; *PINK1*, PTEN, induced putative protein kinase 1; *LRRK2*, Leucine-Rich Repeat Kinase 2; AR, Autosomal Recessive; AD, Autosomal Dominant; BAC, Bacterial Artificial Chromosome; Tg, transgenic; NTg, non-transgenic.

#### 3.2.1. *SNCA* (PARK1, 4)

The *SNCA* gene plays an important role in AD PD, with missense mutations and copy number gains (duplication or triplication) being established causes of PD. While pathogenic missense variants in *SNCA* are rare in the general population, duplications and triplications are also rare but more frequent, with approximately 60 reported families to date (90).

*SNCA* has six exons that encode alpha-synuclein, a 140-amino acid cytoplasmic protein highly abundant in neurons, particularly in the substantia nigra pars compacta (SNc), where it regulates dopamine neurotransmission (83, 91). Pathogenic *SNCA* variantscan lead to cytoplasmic accumulation of alpha-synuclein, promoting oxidative stress and metabolic dysfunction in the SNc (92).

Specific pathogenic variants in the *SNCA* that are associated with PD. Parkinson’s disease-1 (PARK1) results from a point pathogenic variant (missense) in *SNCA*, while *SNCA* gene duplication and triplication lead to Parkinson’s disease-4 (PARK-4) (93). To date, eight pathogenic missense variants have been identified in *SNCA*, all located within the N-terminal amphipathic region of alpha-synuclein. These variants interfere with the alpha-helix-mediated interaction with membranes, contributing to the pathogenesis of PD (94–100).

The most common of these eight missense variants is A53T (18, 80). The number of cases for other missense variants (A30G, A30P, E46K, H50Q, G51D, A53E, and A53V) are small, and some, like H50Q, are not significantly enriched in cases compared to controls (18). Therefore, sufficient evidence may not exist to classify all of them as pathogenic variants (18).

PD patients with *SNCA* gene pathogenic variants typically exhibite earlier age of disease onset than iPD, rapid disease progression, positive response to levodopa treatment, and often present with prominent NMS (101).

This review included six studies, two animal models and four clinical studies (all case series/reports), that investigated *SNCA* pathogenic variants and their potential association with pain. Out of the initially identified articles, 61 were excluded as they did not mention pain or other phenotypes related to *SNCA* pathogenic variants.

Regarding animal studies, Vivacqua et al. reported that higher levels of alpha-synuclein expression in spinal cord areas known to be involved in pain modulation and transmission (84). Another study showed that *PINK1*−/− *SNCA* A53T double mutant mice, which develop a PD-like disease, exhibited a loss of thermal sensitivity as an initial sign of sensory dysfunction (85).

Among clinical studies, one case report study described a novel *SNCA* missense pathogenic variant, G51D, in a patient with lower extremity pain among her symptoms (29). Additionally, two other case reports detailed dorsal and upper back pain in carriers with *SNCA* duplication and triplication (19, 30).Another case report described painful dystonic flexion of the toes while walking in a patient with H50Q variant, although this variant’s pathogenicity remains under debate (28).

#### 3.2.2. *PRKN* (PARK2)

The *PRKN* gene is associated with an AR form of the disorder (79, 102, 103). Homozygous and compound-heterozygous pathogenic variants in *PRKN* are causative of PD, while heterozygous pathogenic variants may predispose to PD symptoms with low penetrance, making them potential genetic risk factors (104, 105).

*PRKN* comprises12 exons and encodes Parkin, a 465 amino acid protein (79, 106). Parkin is widely expressed in human tissues, with significant abundance in the brain, especially the substantia nigra pars compacta (SNc) (106).

Approximately 60 *PRKN* pathogenic and non-pathogenic variants have been identified, including deletions and duplications, which can complicate *PRKN* genotyping (107, 108). According to published data, up to 18% of EOPD patients globally and 27.6% of AR families carry *PRKN* pathogenic variants (23, 109). Among PD-*PRKN* patients, exon 3 deletion is the most frequent pathogenic variant (17). All *PRKN* pathogenic variants result in the loss of Parkin function, leading to a loss of Ubiquitin E3 ligase activity and subsequent neurodegeneration (110).

Monogenic PD associated with *PRKN* typically presents with early onset, slow symptom progression, and a positive response to dopaminergic treatment but is often accompanied by complications such as dystonia and prominent freezing of gait (111, 112).

Sixteen studies in this review provided information on the association between *PRKN* pathogenic variants and pain, including clinical data. These studies included eight case series/reports, three cross-sectional, three case–control studies, and two cohorts. One hundred fifty-six eight clinical studies and one animal study were excluded because they did not mention pain as a symptom or did not involve carriers pathogenic variant carriers.

A cohort study has reported painful limb dystonia as a symptom among *PRKN* pathogenic variant carriers (45). Besides, another cohort study described eight cases with lower limb dystonia activated by walking, Three of those cases also presented with lower limb pain unrelated to dystonic spasms (46).

A case–control study mentioned painful dystonia among three PRKN missense pathogenic carriers and general pain in another missense carrier, while one case report study described painful dystonic posture during off phases in a homozygous exon 3 deletion patient (31, 42). Three case reports described painful foot dystonia among deletion carriers, with four patients in one study also experiencing LBP (32, 33, 37).

Notably, a case series reported 24% dystonia among the patients but did without specifying whether they were *PRKN* pathogenic variant carriers (34). A case–control study reported “other symptoms” including pain and dystonia, as an onset symptoms in 43% of *PRKN* carriers compared to 13% of non-carriers (44).

Musculoskeletal pain has also been associated with *PRKN* pathogenic variants (R275W, exon 3 duplication and homozygous deletion) in two studies (40, 41).

In a cross-sectional study, a carrier of *PRKN* exon 9 deletion reported experiencing pain as a symptom, although the specific type of pain was not described (40). Another cross-sectional study noted painful contractions as an onset symptom in a patient with exon 3 deletion (41). Additionally, a homozygous *PRKN* pathogenic variant carrier required hospitalization due to painful OFF periods (36).

Sensory symptoms and signs, such as tingling sensation and a significant decrease in Sural Sensory Nerve Action Potential (SNAP) amplitude, were reported in two studies (35, 39). Besides, a case–control study observed a sensory gain in cold and hot pain thresholds among carriers (43).

On the other hand, there was a study screened 145 PD patients for *LRRK2* pathogenic variant, 19 of whom carried a *PRKN* pathogenic variant. They reported their clinical data, including pain, and no specific mention of pain was reported for any of the pathogenic variant carriers (38).

#### 3.2.3. *PINK1* (PARK6)

Pathogenic variants in the *PINK1* gene are the second most common cause of AR EOPD (79). *PINK1* comprises 6 exons encoding PTEN-induced putative kinase 1, a 581 amino acid serine/threonine kinase (113). In normal conditions, wild-type *PINK1* plays a protective role against neuronal apoptosis in neural cell lines. However, pathogenic variants associated with PARK6 disrupt this protective function, leading to the degeneration of dopaminergic neurons (114). Most *PINK1* pathogenic variants are located in exon 7, with Q456X variant being the most frequent (79).

Individuals with *PINK1* monogenic PD typically have an onset age of around 32 years and experience slow disease progression, often with a favorable response to levodopa treatment and sleep benefits (115).

Thirteen studies have investigated the potential relationship between pain and *PINK1* pathogenic variants. These studies include two animal studies and eleven clinical research articles (seven case series/reports, one cross-sectional study, and three case–control studies). Forty-one clinical studies were excluded from the analysis because they did not mention pain as a symptom.

Regarding animal studies, a study found that thermal withdrawal latencies were significantly shorter in *PINK1*−/− rats than in wild-type rats over time, indicating altered pain responses (86). The second team used a rat model with neuropathic pain to investigate the role of *PINK1* and observed increased expression of *PINK1* in pain-related areas compared to control rats (87).

A case–control study reported painful episodes of torticollis and painful dystonia in a homozygous Q456X *PINK1* pathogenic variant carrier (54). Similarly, Zadikoff et al. described a homozygous Q456X *PINK1* pathogenic variant carrier who frequently experienced back and limb pain and painful wearing-off dystonia (47).

Multiple case reports, one case–control, and one cross-sectional study support the observation that pain is frequently encountered in patients with *PINK1* pathogenic variants, often manifesting in various body regions, particularly the neck, back, and shoulders. However, these studies did not provide detailed descriptions of the specific type and characteristics of the reported pain (20, 48–50, 53, 55).

Additionally, a case report study highlighted a patient with a novel homozygous nonsense *PINK1* pathogenic variant. This patient exhibited an urge to move her lower limbs accompanied by painful paresthesia and a sensation of distal cramping (51).

On the other hand, one case report (a homozygous Q456X carrier) and one case–control study specifically assessed pain symptoms in individuals carrying *PINK1* mutations, but neither of them reported pain as a symptom among the *PINK1* carriers (38, 44). In the case–control study, the prevalence of pain among *PINK1* carriers was 0%, while it was 13% in the non-carrier group, it was 13, and 43% in *PRKN* carriers (44).

#### 3.2.4. *LRRK2* (PARK8)

Pathogenic variants in the *LRRK2* gene are the most common genetic cause of AD PD (24, 116), affecting both familial and sporadic forms of the disease (117). The *LRRK2* gene is a large gene consisting of 51 exons and encodes a 2,527 amino acid cytoplasmic protein called leucine-rich repeat kinase 2 (118). One of the critical functions of *LRRK2* is its regulation of protein synthesis through the miRNA pathway, and impairment in this pathway has been implicated in *LRRK2*-related pathogenesis (119).

More than 40 pathogenic variants have been identified in the *LRRK2* gene among PD patients, with eight of them known to cause PD (93). Among them, the most common and well-characterized *LRRK2* pathogenic variant is G2019S, with a prevalence ranging from 0 to 42% depending on ethnicity, followed by R1441C (120, 121).

*LRRK2*-PD patients typically presents as a LOPD, often respond well to levodopa treatment, and have fewer NMS than iPD cases (122, 123).

Among the identified studies involving *LRRK2*-associated PD patients, 18 included information on pain symptoms, while 124 studies did not mention pain or other sensory symptoms and were excluded from the analysis. Of the 18 studies, two were animal studies, and the remaining 16 were clinical consisting of five case series/reports, five cross-sectional studies, five case–control studies, and one cohort study.

Evidence from mouse studies investigating the association between *LRRK2* and pain observed similar pain sensitivity than controls without developing sensory deficits (88, 89).

Multiple studies have described the presence of painful dystonia in different cohorts of *LRRK2* pathogenic variant carriers. Three studies reported painful dystonia among G2019S pathogenic variant carriers. A case–control study reported that 42% of carriers experienced painful foot dystonia during the “OFF period” (64). Another case report described a patient who reported painful foot dystonia (58). Furthermore, a study reported painful cervical dystonia in one individual, which showed a positive response to levodopa treatment (57).

Severe wearing-off and dyskinesia with off-time pain have been reported in a *LRRK2* pathogenic variant carrier (59). Unspecified pain and joint pain have been reported as onset symptoms by three PD patients, all carriers of pathogenic variants, two G2019S and one R1441C (21, 61, 69). A case–control study found that pain is one of the most common NMS experienced by PD patients with *LRRK2* pathogenic mutations, affecting over half of the subjects (65). In a cross-sectional study, pain was observed in five R1441C carriers but only in one non-carrier, although the difference was not statistically significant (*p* = 0.155). The specific type and characteristics of the reported pain were not described in detail (62).

Notably, a case report documented a patient with severe and painful ON-dystonia who carried a *LRRK2* N1437H variant which is not recognized among the established pathogenic variants (56). Furthermore, McGill test recorded neuropathic disturbances were reported for *LRRK2* pathogenic variant carriers with a mean of 8.3 8.3 ± 14 compared to 0 for non-carriers. Additionally, *LRRK2* mutation carriers displayed a higher heat pain threshold compared to non-carriers (44.1 ± 4.82 vs. 40.6 ± 4.5°C, *p* = 0.058), suggesting a clear difference in terms of pain perception (60).

The final five included studies for this gene reported pain among individual carrying *LRRK2* pathogenic variants. However, after analysis, no statistically significant difference in the prevalence of pain emerged between the carrier and non-carrier groups (52, 63, 66–68).

#### 3.2.5. *PARK7* (DJ-1)

*PARK7* pathogenic variants are associated with AR PD and are relatively uncommon, constituting approximately 1 to 2% of EOPD cases (124). The *PARK7* gene is comprised of 8 exons, with the initial two being noncoding, and it encodes DJ-1, a 189 amino acid protein which exhibits neuroprotective and antioxidant properties (125, 126). Pathogenic variants within *PARK7* result in the production of a mutated DJ-1 protein characterized by reduced activity due to misfolding (127, 128).

Individual carrying *PARK7* pathogenic variant typically experience disease onset at an average age of 27 years and often exhibit prominent NMS, including mental health disorders and cognitive decline. Dystonia is highly prevalent, affecting approximately 73% of those with DJ-1 pathogenic variant (129).

Among the 26 studies examining *PARK7* pathogenic variants, only one case series/report study mentioned the presence of pain.

Djarmati et al. conducted a screening of 75 unrelated PD patients and identified one individual carrying a heterozygous deletion of exon 5 in *PARK7*. Among the 75 cases, three individuals presented pain as an onset symptom (4%); however, the authors did not specify whether the *PARK7* pathogenic variant carrier was one of the three PD patients reporting pain (34).

#### 3.2.6. *VPS35* (PARK17)

*VPS35* is responsible for encoding the vacuolar protein sorting ortholog 35, which is a critical component of a large complex involved in the transportation of proteins from endosomes to the trans-Golgi network. Pathogenic variants in *VPS35* were initially identified in 2011 and represent a rare cause of AD LOPD (18, 130).

Exome analysis has revealed that the D620N is the sole confirmed pathogenic variant associated wit PD thus far (131). Monogenic PD linked to *VPS35* exhibits high heritability but low penetrance. The clinical phenotype of *VPS35*-related PD closely resembles that of iPD, although the average age of onset is typically around 50 years old (132).

Among the twelve studies conducted on individual carrying *VPS35* variants, 67 patients were included, all of whom were heterozygous carriers (18). Out of these 67 heterozygous patients, who presented a total of 10 different potentially disease-causing variants, 50 (75%) carried the pathogenic D620N variant (18). While these studies did report various symptoms in these patients, NMS were the least commonly reported (6.2%) and none of the studies specifically mentioned the presence of pain (18).

#### 3.2.7. GBA1

Pathogenic variants in the *GBA1* gene are not considered causative for PD, but they represent the most prevalent genetic susceptibility factor for the development of the disease (133, 134). While *GBA1* pathogenic variants do not exhibit complete penetrance, heterozygote carriers face a fivefold increased risk of developing PD, while homozygotes have a 10- to 20-fold elevated risk (135). The penetrance of *GBA* pathogenic variant carriers to develop PD has been estimated as 13.7% by the age of 60 years and 29.7% by the age of 80 years (135).

Furthermore, due to their higher frequency in most PD populations compared to known monogenic PD genes such as *LRRK2*, *SNCA*, and *PRKN* (136), *GBA1* pathogenic variants are regarded as the most significant genetic risk factor for PD (137). Recent genome-wide association studies have confirmed that approximately 8–12% of PD patients carry *GBA1* pathogenic variants (138).

The *GBA1* gene, located on chromosome 1q22, encodes the enzyme glucocerebrosidase, and is associated with AR Gaucher disease (GD) (136). Approximately 130 *GBA1* pathogenic variants have been reported in PD patients (27, 139). Similar to GD, L444P and N370S are the two most frequent pathogenic variants. Severe pathogenic variants such as L444P are associated with a higher risk of developing PD, earlier age of onset, and more severe motor and NMS (140).

We included seven articles (comprising four case–control studies, two case series/reports, and one cross-sectional study). Eighteen articles were excluded because they did not mention pain among the reported symptoms.

While no distinctive symptoms have been reported to differentiate *GBA1* pathogenic variant carriers from individuals with iPD (141), pain appears to be an exception. Some patients with GD develop progressive parkinsonian symptoms (142), and notably, pain has been more frequently reported as an initial symptom in *GBA1*-PD patients compared to individuals with iPD.

Shoulder pain and LBP (76), unexplained pain (58% vs. 10%, *p* = 0.005) (75), and cramp-like pain as the primary source of disability at a young age (72) have all been reported more frequently among *GBA1* pathogenic variant carriers compared to non-carriers. In a case series pain was identified as the most bothersome symptom in 12 out of 15 *GBA1*-PD patients also reporting rigidity and stiffness, often accompanied by pain. One patient described painful dyskinesia as the most bothersome symptom, and two reported pain-related sleep problems (71).

In a cross-sectional study, pain was reported more frequently as an initial symptom in the *GBA1-*PD compared to the iPD 10.3 vs. (3.0%) (*p* = 0.039), with four patients reporting shoulder pain as their initial symptom. The most significant finding of this study is that the presenting symptoms of PD are similar in *GBA1* carriers and non-carriers for all parameters except for pain (73).

On the other hand, two case–control studies mentioned that *GBA1*-PD patients experienced bodily pain among their symptoms, although no statistically significant differences were reported between *GBA1*-PD and the iPD group (*p* = 0.7) (67, 74).

Considering that symptoms tend not be more severe among patients carrying pathological variants like L444P, it becomes intriguing to explore whether pain is more closely associated with severe variants (137). Contrary to this argument, based on existing studies, while two patients with N370S variant (considered mild) reported cramp-like pain (72), 49 patients with L444P variant (classified as severe) found no significant differences in terms of bodily pain compared to individuals with iPD (67).

#### 3.2.8. *ATP13A2* (PARK9)

Pathogenic variants in the *ATP13A2* gene are responsible for Kufor-Rakeb syndrome (KRS), an AR atypical form of PD (143). The *ATP13A2* gene consists of 29 exons and encodes a protein of 1,180 amino acids (144). The *ATP13A2* protein plays a role in reducing intracellular concentrations of manganese ions (Mn2+), thereby offering protection against apoptosis (145). Pathogenic variants in *ATP13A2* lead to disruptions in the proteasomal pathway and premature degradation of *ATP13A2* mRNA, contributing to the development of KRS (146).

Since the discovery of *ATP13A2* pathogenic variants in 2006 (144), only a limited number of studies have been conducted and published. Among the 16 studies we assessed, one cross-sectional study and one case series/report did mention pain as a symptom, while the remaining 14 studies did not mention pain or other associated phenotypes and were consequently excluded.

In one cross-sectional study, a *ATP13A2* pathogenic variant was identified in two patients who did not have pain as one of their symptoms (38). Additionally, a case report documented arm dystonic posturing as the onset symptom in a homozygous patient with 2822delG variant who was unresponsive to anticholinergics and levodopa; however this variant has not yet been definitively established as a pathogenic variant for PD (70).

#### 3.2.9. *DNAJC6* (PARK19A, b)

*DNAJC6*, located on 1p31.3, encodes auxilin, and its loss of function can lead to EOPD (147). In animal studies, the absence of auxilin has been linked to synaptic vesicle endocytosis disruptions, which have adverse effects on synaptic neurotransmission, homeostasis, and signaling (148). However, the precise mechanism by which auxilin deficiency induces dopaminergic neurodegeneration and unusual neurological symptoms remains incompletely understood (148).

Homozygous pathogenic variants in *DNAJC6* are responsible for atypical parkinsonism, exhibiting AR inheritance pattern (149, 150). PARK19A is characterized by onset in the first or second decade of life and rapid disease progression, while PARK-19B onset occurs between the third and fifth decades, featuring a slower progressive course, and similar features to classic iPD (149–151).

Three separate case-report studies identified Juvenile-onset PD, PARK-19A, among patients with homozygous pathogenic variant in the *DNAJC6* gene, including two loss-of-function and one nonsense variant; however, none of these cases reported pain as a symptom (149, 150, 152). Another study reported homozygous pathogenic variants in two unrelated families with PARK-19B and no instances of pain were among their reported symptoms (151).

Finally, in a comprehensive analysis utilizing whole exome sequencing *DNAJC6* potential pathogenic variants were explored in 6 juvenile parkinsonism patients. Homozygous nonsense R256* *DNAJC6* pathogenic variants were confirmed for all affected children and none of them reported pain among their symptoms (153).

#### 3.2.10. *FBXO7* (PARK15)

*FBXO7* a gene comprising ten exons is located on chromosome 22q12.3, encodes a member of the F-box protein family known as F-Box Protein 7, characterized by an approximately 40 amino acid motif (154). Pathogenic variants in *FBXO7* are responsible for an AR parkinsonian syndrome. The typical presenting symptoms include bradykinesia and tremor, and patients affected by this disorder frequently exhibit pyramidal signs, dysarthria, and dyskinesia (155).

To date, eight studies have identified cases carrying *FBXO7* variants, predominantly associated with an early-onset parkinsonian and pyramidal syndrome (155–161). Notably, only one study reported a classical PD presentation in two siblings, caused by a new *FBXO7* pathogenic variant, L34R (162). None of these studies discuss or mention pain as one of the associated symptoms.

#### 3.2.11. *SYNJ1* (PARK20)

*SYNJ1*, located on 21q22.11 and comprised of 33 exons, encodes Synaptojanin 1 protein. Pathogenic variant in *SYNJ1* Are associated to AR EOPD (163).

Remarkably, independently and simultaneously, two studies identified the same homozygous missense pathogenic variant in the *SYNJ1* gene, R258Q. In both studies affected patients were thoroughly screened for all known genes, and R258Q *SYNJ1* was the sole pathogenic variant identified. Two affected siblings in each study suffered from EOPD, and none of them mention pain among their symptoms (164, 165).

## 4. Discussion

This review was conducted to assess the presence of pain in patients with monogenic PD-related pathogenic variants, encompassing genes such as *SNCA, PRKN, PINK1, LRRK2, ATP13A2, PARK7, VPS35, GBA1, DNAJC6, FBXO7,* and *SYNJ1*. The central findings of this review offer valuable insights into the connection between specific gene pathogenic variants and the occurrence of pain in individuals with PD.

**Figure 2:**
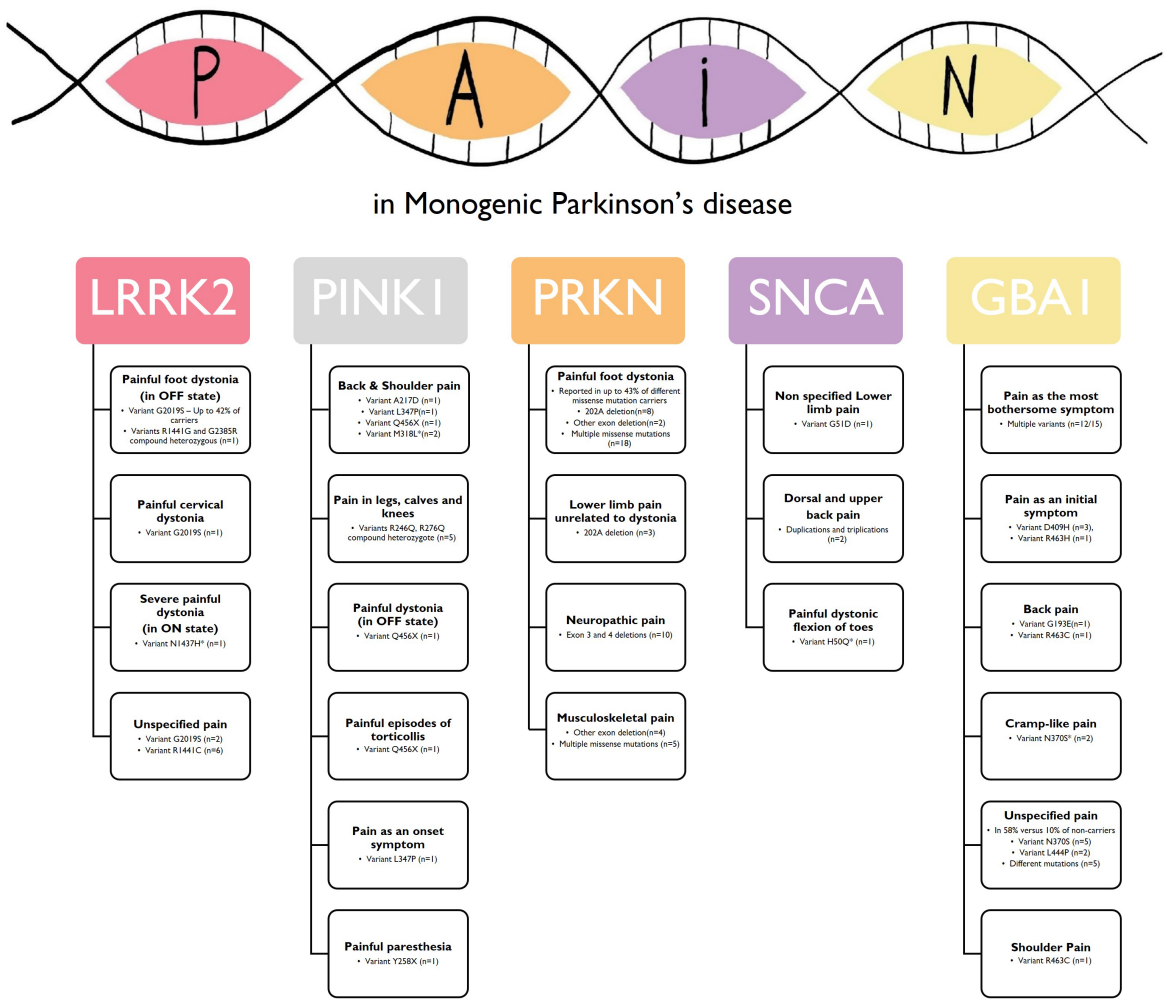
Common pain subtypes linked with Monogenic Parkinson’s disease. This figure illustrates the most commonly pain subtypes reported in individuals with monogenic forms of Parkinson’s disease (PD) associated with specific genes. The figure provides an overview of the pain profiles observed in relation to each gene pathogenic variant using the descriptors used in the original articles. *LRRK2*, Leucine-Rich Repeat Kinase 2; *PINK1*, PTEN induced putative protein kinase 1; *PRKN*, Parkin RBR E3 Ubiquitin Protein Ligase; *SNCA*, alpha-synuclein; *GBA*, Glucosylceramidase – *Variants that are not yet established as pathogenic for PD.

As a summary: (1) for the *SNCA* gene, two point mutations were associated with lower extremity pain and painful foot dystonic flexion while walking (28, 29). Gene duplications and triplications were also linked to dorsal and upper back pain (19, 30); (2) *PRKN* carriers reported painful lower limb dystonia and lower back pain as prominent symptoms (31–33, 36, 37, 42, 45, 46). Additionally, musculoskeletal pain, sensory loss, tingling sensation, and reduced SNAP amplitude suggested a central origin for abnormal sensitivity in *PRKN* pathogenic variant carriers (35, 39–41, 43); (3) Pain was observed in *PINK1* pathogenic variants. Homozygote carriers of the Q456X as the most frequent pathogenic variant experienced painful dystonia (47, 54), and pain was reported in various body parts, with a preference for the neck, back, and shoulders (20, 48–50, 53, 55). *PINK1* pathogenic variants were also associated with abnormal central somatosensory processing (51); (4) The *LRRK2* pathogenic variants are associated with pain, with painful dystonia reported in G2019S carriers (57, 58, 64) and G2019S and R1441C carriers reporting unspecified joint pain as their onset symptom (21, 61, 69). Multiple studies indicated that *LRRK2* pathogenic variant carriers experienced different types of pain as part of their symptoms (52, 56, 59, 60, 62, 63, 66–68); (5) Limited studies have assessed pain in *ATP13A2* pathogenic variant carriers. However, pain was reported in a few cases, suggesting a potential link between *ATP13A2* and pain (70); (6) Among *GBA1* pathogenic variant carriers, pain was reported as one of the most prevalent early symptoms, with some patients exclusively experiencing shoulder pain as an initial presentation (73). A case series study found that almost all *GBA1*-PD patients reported pain as their most bothersome symptom (71). Glucosylceramide accumulation, associated with *GBA1* pathogenic variants, may contribute to PD-associated sensory neuropathies and pain (166). Low *GBA1* activity has also been observed in PD patients without *GBA1* pathogenic variants, indicating its involvement in developing or progressing PD-associated sensory neuropathy (167). It would be indeed interesting to explore whether pain is more associated with severe pathological variants in PD-related genes. However, based on the available studies, there does not appear to be a significant difference in the prevalence of pain between individuals with severe pathological variants and those with iPD (67, 137); (7) No results were available regarding pain in *PARK7*, *VPS35, DNAJC6, FBXO7,* and *SYNJ1* pathogenic variants. The most common pain subtypes linked with Monogenic Parkinson’s disease are summarized in Figure 2.

The findings discussed in this review provide valuable insights into the connection between specific monogenic variants in PD-related genes and pain in PD. Certain genes roles, including *SNCA, PRKN, PINK1,* and *LRRK2*, have been extensively studied providing potential perspectives into the underlying mechanisms of pain in PD.

In the case of *SNCA*, animal studies have indicated that abnormal pain in PD may be attributed to pathological changes related to alpha-synuclein- presence in unmyelinated areas of the spinal cord (84, 85). Clinical studies further support the presence of various pain manifestations in *SNCA* pathogenic variant carriers, such as painful dystonic flexion while walking and dorsal and upper back pain (19, 28, 29).

Similarly, *PRKN* pathogenic variant carriers have been found to experience painful lower limb dystonia and lower back pain, accompanied by reduced SNAP amplitude (31–33, 35–37, 39–43, 45, 46). These findings suggest the involvement of sensory axonal neuropathy and suggest that reduced SNAP amplitude may serve as a diagnostic indicator for *PRKN*-related PD.

*PINK1* pathogenic variant carriers, they exhibit distinct somatosensory profiles and clinical entities compared to iPD, suggesting a primary hypofunction of nociceptive and non-nociceptive systems in *PINK1*-associated PD (43). Studies have proposed that specific *PINK1* pathogenic variants, such as the L347P, may be associated with pain in PD patients (20). Moreover, abnormalities in nociceptive processing have been reported in *PINK1* pathogenic variant carriers, indicating a potential role of abnormal central somatosensory processing in pain generation (43). Interestingly, these abnormalities seem to lead to hypoalgesia rather than hyperalgesia, contrasting with the findings in sporadic PD cases (168). The study of E3 ligase dysfunction has provided insights into the pathophysiology of PD, particularly about the *PRKN* gene (169). The single-base pair deletion in *PRKN* observed in four brothers with refractory back pain may be attributed to a lack of E3 activity, potentially contributing to lower back pain in PD patients (37). E3 ligases play a crucial role in the ubiquitin-proteasome pathway involved in protein turnover, and dysfunction in this pathway has been implicated in PD (169).

Additional research is needed to better understand the connection between *LRRK2* and pain as while multiple clinical studies have suggested that individuals with LRRK2 pathogenic variants experience different pain types (21, 58, 64, 88), animal models findings also suggest that pain sensitivity remains unchanged in the presence of *LRRK2* pathogenic variants (89, 123).

In the context of other monogenic variants, such as *ATP13A2, PARK7, VPS35*, *DNAJC6, FBXO7* and *SYNJ1* the current literature provides inconclusive results or insufficient data regarding their association with pain in PD. Additional studies are required to clarify the potential links between these genes and pain symptoms in PD patients.

Genetic associations with *GBA1* variants have demonstrated an influence the occurrence of pain in PD. Patients carrying *GBA1* variants have reported higher rates of pain compared to non-carriers (71, 73). Recent studies suggest that approaches targeting glucocerebrosidase activity or refolding may reduce PD pain and sensory loss (166). Even in PD patients without *GBA1* variants, low *GBA1* activity has been observed, indicating a prevalent loss of *GBA1* function that may contribute to developing or progressing PD-associated sensory neuropathy (135). These findings suggest that elevated levels of glucosylceramides may underlie sensory neuropathies characterized by the loss of thermal sensation and mechanical hypersensitivity in PD patients, irrespective of the presence of chronic pain.

Overall, this comprehensive review underscores the complex relationship between monogenic pathogenic variants in PD-related genes and the presence of pain in PD. To advance our understanding of the underlying mechanisms and identify potential targets for the treatment of pain in PD further investigations are essential. While this review provides a solid foundation for future research, it also sheds light on several limitations that require attention. The absence of pain assessment in numerous studies and the lack of detailed pain characteristics impede a comprehensive understanding of pain in monogenic PD-related pathogenic variants. Furthermore, the predominance of case series/reports and the limited information available for specific gene pathogenic variants underscores the necessity for more robust studies with larger sample sizes and systematic evaluation of pain symptoms. It is because of these limitation specific frequencies or data about the prevalence of pain in monogenic forms of PD remain unclear. Addressing this knowledge gap is of paramount importance and needs the implementation of more focused and structured study designs regarding pain in PD. Finally, gaining a deeper understanding of pain as a potential prodromal symptom in monogenic PD could provide insights into early indicators and predictive markers, allowing for more timely and targeted interventions.

## 5. Conclusion

In conclusion, the existing evidence suggests that specific types of pain are commonly observed in individuals with monogenic forms of PD, particularly those associated with *SNCA, PRKN, PINK1, LRRK2*, and *GBA1* genes. Pain in PD can potentially serve as a clinical marker, sometimes as a prodromal symptom as in individuals with *PRKN* and *GBA1* pathogenic variants, but also as a potential marker of progression other genes pathogenic variants.

Given the subjective nature of pain, its effective management requires standardized and objective standards of care. Future investigations should prioritize the collection of high-quality, standardized pain data, to enable direct comparison across studies and facilitate large-scale meta-analyses. Establishing connections between genetic profiles with pain symptoms could have significant clinical implications, such as guiding the selection of diagnostic tests, facilitating patient stratification for clinical trials, and ultimately enabling personalized treatment approaches for individuals with monogenic PD.

## Author contributions

VB contributed to the study’s design, planning, supervision, and manuscript review. PA and CT-C were involved in the planning, literature search, and manuscript writing. BA participated in the study’s original design and search strategy. All authors critically reviewed and approved the final version of the manuscript.

## References

[1] Ford B. Pain in Parkinson’s disease. Mov Disord. (2010) 25:S98–S103. doi: 10.1002/mds.22716 20187254

[2] Ha AD Jankovic J. Pain in Parkinson’s disease. Mov Disord. (2012) 27:485–91. doi: 10.1002/mds.23959 21953990

[3] Chaudhuri KR Rizos A Trenkwalder C Rascol O Pal S Martino D. King’s Parkinson’s disease pain scale, the first scale for pain in PD: an international validation: King’s PD pain scale validation. Mov Disord. (2015) 30:1623–31. doi: 10.1002/mds.26270, PMID: 26096067

[4] Broen MPG Braaksma MM Patijn J Weber WEJ. Prevalence of pain in Parkinson’s disease: a systematic review using the modified QUADAS tool: prevalence of pain in Parkinson’s disease. Mov. Disord. (2012) 27:480–4. doi: 10.1002/mds.24054 22231908

[5] Defazio G Gigante A Mancino P Tinazzi M. The epidemiology of pain in Parkinson’s disease. J Neural Transm. (2013) 120:583–6. doi: 10.1007/s00702-012-0915-7 23208197

[6] Toda K Harada T. Prevalence, classification, and etiology of pain in Parkinson’s disease: association between Parkinson’s disease and fibromyalgia or chronic widespread pain. Tohoku J Exp Med. (2010) 222:1–5. doi: 10.1620/tjem.222.1 20805678

[7] Ford B. Pain in Parkinson disease: the hidden epidemic. Nat Rev Neurol. (2009) 5:242–3. doi: 10.1038/nrneurol.2009.50, PMID: 19488080

[8] Mao CJ Chen JP Zhang XY Chen Y Li SJ Li J. Parkinson’s disease patients with pain suffer from more severe non-motor symptoms. Neurol Sci. (2015) 36:263–8. doi: 10.1007/s10072-014-1942-y 25192663

[9] Quittenbaum BH Grahn B. Quality of life and pain in Parkinson’s disease: a controlled cross-sectional study. Parkinsonism Relat Disord. (2004) 10:129–36. doi: 10.1016/j.parkreldis.2003.12.001, PMID: 15036166

[10] Rodríguez-Violante M Alvarado-Bolaños A Cervantes-Arriaga A Martinez-Martin P Rizos A Chaudhuri KR. Clinical determinants of Parkinson’s disease-associated pain using the King’s Parkinson’s disease pain scale. Mov Disord Clin Pract. (2017) 4:545–51. doi: 10.1002/mdc3.12469, PMID: 30363423 PMC6174437

[11] Tinazzi M. Pain and motor complications in Parkinson’s disease. J Neurol Neurosurg Psychiatry. (2006) 77:822–5. doi: 10.1136/jnnp.2005.079053, PMID: 16549416 PMC2117476

[12] Buhmann C Kassubek J Jost WH. Management of Pain in Parkinson’s disease. J Parkinsons Dis. (2020) 10:S37–48. doi: 10.3233/JPD-202069 32568113 PMC7592654

[13] Tueth LE Duncan RP. Musculoskeletal pain in Parkinson’s disease: a narrative review. Neurodegener Dis Manag. (2021) 11:373–85. doi: 10.2217/nmt-2021-0011, PMID: 34410146 PMC8515213

[14] Stamey W Davidson A Jankovic J. Shoulder pain: a presenting symptom of Parkinson disease. JCR J Clin Rheumatol. (2008) 14:253–4. doi: 10.1097/RHU.0b013e3181826d43 18766134

[15] Cleeves L Findley L. Frozen shoulder and other shoulder disturbancies in Parkinson’s disease. J Neurol Neurosurg Psychiatry. (1989) 52:813–4. doi: 10.1136/jnnp.52.6.813-b, PMID: 2746285 PMC1032056

[16] Barbeau A Roy M. Familial subsets in idiopathic Parkinson’s disease. Can J Neurol Sci J Can Sci Neurol. (1984) 11:144–50. doi: 10.1017/S0317167100046308 6713312

[17] Kasten M Hartmann C Hampf J Schaake S Westenberger A Vollstedt EJ. Genotype-phenotype relations for the Parkinson’s disease genes *Parkin*, *PINK1*, *DJ1*: MDSGene systematic REVIEW: MDSGene REVIEW: *Parkin*, *PINK1*, *DJ1*. Mov Disord. (2018) 33:730–41. doi: 10.1002/mds.27352 29644727

[18] Trinh J Zeldenrust FMJ Huang J Kasten M Schaake S Petkovic S. Genotype-phenotype relations for the Parkinson’s disease genes *SNCA*, *LRRK2*, VPS35: MDSGene systematic review: MDSGene systematic review: *SNCA*, *LRRK2*, *VPS35*. Mov Disord. (2018) 33:1857–70. doi: 10.1002/mds.27527, PMID: 30357936

[19] Perandones C Giugni JC Calvo DS Raina GB De Jorge LL Volpini V. Mosaicism of alpha-synuclein gene rearrangements: report of two unrelated cases of early-onset parkinsonism. Parkinsonism Relat Disord. (2014) 20:558–61. doi: 10.1016/j.parkreldis.2013.11.014, PMID: 24552873 PMC4197942

[20] Rogaeva E Johnson J Lang AE Gulick C Gwinn-Hardy K Kawarai T. Analysis of the *PINK1* gene in a large cohort of cases with Parkinson disease. Arch Neurol [Internet]. (2004) 61:1898–904. doi: 10.1001/archneur.61.12.1898 15596610

[21] Gatto EM Parisi V Converso DP Poderoso JJ Carreras MC Martí-Massó JF. The LRRK2 G2019S mutation in a series of Argentinean patients with Parkinson’s disease: clinical and demographic characteristics. Neurosci Lett. (2013) 537:1–5. doi: 10.1016/j.neulet.2013.01.011, PMID: 23340200

[22] Tricco AC Lillie E Zarin W O’Brien KK Colquhoun H Levac D. PRISMA extension for scoping reviews (PRISMA-ScR): checklist and explanation. Ann Intern Med. (2018) 169:467–73. doi: 10.7326/M18-0850, PMID: 30178033

[23] Domingo A Klein C. Genetics of Parkinson disease. Handb Clin Neurol. (2018):211–27. doi: 10.1016/B978-0-444-63233-3.00014-2 29325612

[24] Cook L Schulze J Verbrugge J Beck JC Marder KS Saunders-Pullman R. The commercial genetic testing landscape for Parkinson’s disease. Parkinsonism Relat Disord. (2021) 92:107–11. doi: 10.1016/j.parkreldis.2021.10.001 34696975 PMC8633166

[25] Jia F Fellner A Kumar KR. Monogenic Parkinson’s disease: genotype, phenotype, pathophysiology, and genetic testing. Genes. (2022) 13:471. doi: 10.3390/genes13030471, PMID: 35328025 PMC8950888

[26] Zimprich A Benet-Pagès A Struhal W Graf E Eck SH Offman MN. A mutation in VPS35, encoding a subunit of the Retromer complex, causes late-onset Parkinson disease. Am J Hum Genet. (2011) 89:168–75. doi: 10.1016/j.ajhg.2011.06.008, PMID: 21763483 PMC3135812

[27] Zhang Y Shu L Sun Q Zhou X Pan H Guo J. Integrated genetic analysis of racial differences of common GBA variants in Parkinson’s disease: a meta-analysis. Front Mol Neurosci. (2018) 11:43. doi: 10.3389/fnmol.2018.00043, PMID: 29527153 PMC5829555

[28] Appel-Cresswell S Vilarino-Guell C Encarnacion M Sherman H Yu I Shah B. Alpha-synuclein p.H50Q, a novel pathogenic mutation for Parkinson’s disease: α- Synuclein p.H50q, a novel mutation for Pd. Mov Disord. (2013) 28:811–3. doi: 10.1002/mds.25421 23457019

[29] Lesage S Anheim M Letournel F Bousset L Honoré A Rozas N. G51D α-synuclein mutation causes a novel parkinsonian-pyramidal syndrome: *SNCA* G51D in parkinsonism. Ann Neurol. (2013) 73:459–71. doi: 10.1002/ana.23894, PMID: 23526723

[30] Byers B Cord B Nguyen HN Schüle B Fenno L Lee PC. *SNCA* triplication Parkinson’s Patient’s iPSC-derived DA neurons accumulate α-Synuclein and are susceptible to oxidative stress. PLoS One. (2011) 6:e26159 doi: 10.1371/journal.pone.0026159 22110584 PMC3217921

[31] Capecci M Passamonti L Annesi F Annesi G Bellesi M Candiano ICC. Chronic bilateral subthalamic deep brain stimulation in a patient with homozygous deletion in the Parkin gene. Mov Disord. (2004) 19:1450–2. doi: 10.1002/mds.20250, PMID: 15390056

[32] Khan NL. Progression of nigrostriatal dysfunction in a parkin kindred: an [18F]dopa PET and clinical study. Brain. (2002) 125:2248–56. doi: 10.1093/brain/awf237 12244082

[33] Dogu O Johnson J Hernandez D Hanson M Hardy J Apaydin H. A consanguineous Turkish family with early-onset Parkinson’s disease and an exon 4 parkin deletion: a Turkish Parkin family. Mov Disord. (2004) 19:812–6. doi: 10.1002/mds.20028 15254940

[34] Djarmati A Hedrich K Svetel M Schäfer N Juric V Vukosavic S. Detection of *Parkin* (*PARK2*) and *DJ1* (*PARK7*) mutations in early-onset Parkinson disease: *Parkin* mutation frequency depends on ethnic origin of patients: MUTATION IN BRIEF. Hum Mutat. (2004) 23:525–5. doi: 10.1002/humu.9240, PMID: 15108293

[35] Ohsawa Y Kurokawa K Sonoo M Yamada H Hemmi S Iwatsuki K. Reduced amplitude of the sural nerve sensory action potential in PARK2 patients. Neurology. (2005) 65:459–62. doi: 10.1212/01.wnl.0000171859.85078.3d 16087916

[36] Khan NL Graham E Critchley P Schrag AE Wood NW Lees AJ. Parkin disease: a phenotypic study of a large case series. Brain. (2003) 126:1279–92. doi: 10.1093/brain/awg142, PMID: 12764051

[37] Nisipeanu P Inzelberg R Abo Mouch S Carasso RL Blumen SC Zhang J. Parkin gene causing benign autosomal recessive juvenile parkinsonism. Neurology. (2001) 56:1573–5. doi: 10.1212/WNL.56.11.1573 11402119

[38] Bouhouche A Tesson C Regragui W Rahmani M Drouet V Tibar H. Mutation analysis of consanguineous Moroccan patients with Parkinson’s disease combining microarray and gene panel. Front Neurol. (2017) 8:567. doi: 10.3389/fneur.2017.00567, PMID: 29163333 PMC5674924

[39] Shyu WC Lin SZ Chiang MF Pang CY Chen SY Hsin YL. Early-onset Parkinson’s disease in a Chinese population: 99mTc-TRODAT-1 SPECT, Parkin gene analysis and clinical study. Parkinsonism Relat Disord. (2005) 11:173–80. doi: 10.1016/j.parkreldis.2004.12.004 15823482

[40] Monroy-Jaramillo N Guerrero-Camacho JL Rodríguez-Violante M Boll-Woehrlen MC Yescas-Gómez P Alonso-Vilatela ME. Genetic mutations in early-onset Parkinson’s disease Mexican patients: molecular testing implications. Am J Med Genet B Neuropsychiatr Genet. (2014) 165:235–44. doi: 10.1002/ajmg.b.32228, PMID: 24677602

[41] Lesage S Magali P Lohmann E Lacomblez L Teive H Janin S. Deletion of the *parkin* and *PACRG* gene promoter in early-onset parkinsonism. Hum Mutat. (2007) 28:27–32. doi: 10.1002/humu.20436, PMID: 17068781

[42] Doherty KM Silveira-Moriyama L Parkkinen L Healy DG Farrell M Mencacci NE. Parkin disease: a Clinicopathologic entity? JAMA Neurol. (2013) 70:571–9. doi: 10.1001/jamaneurol.2013.172, PMID: 23459986 PMC4202385

[43] Gierthmühlen J Schumacher S Deuschl G Fritzer E Klein C Baron R. Somatosensory function in asymptomatic *Parkin-* mutation carriers: sensory symptoms and Parkinson’s disease. Eur J Neurol. (2010) 17:513–7. doi: 10.1111/j.1468-1331.2009.02797.x 19863651

[44] Koziorowski D Hoffman-Zacharska D Sławek J Jamrozik Z Janik P Potulska-Chromik A. Incidence of mutations in the PARK2, *PINK1*, *PARK7* genes in polish early-onset Parkinson disease patients. Neurol Neurochir Pol. (2013) 47:319–24. doi: 10.5114/ninp.2013.36756, PMID: 23986421

[45] Hassin-Baer S Hattori N Cohen OS Massarwa M Israeli-Korn SD Inzelberg R. Phenotype of the 202 adenine deletion in the *parkin* gene: 40 years of follow-up: phenotype of the 202A deletion in the *parkin* gene. Mov Disord. (2011) 26:719–22. doi: 10.1002/mds.23456, PMID: 21506149

[46] Elia AE Del Sorbo F Romito LM Barzaghi C Garavaglia B Albanese A. Isolated limb dystonia as presenting feature of Parkin disease. J Neurol Neurosurg Psychiatry. (2014) 85:827–8. doi: 10.1136/jnnp-2013-307294, PMID: 24659796

[47] Zadikoff C Rogaeva E Djarmati A Sato C Salehi-Rad S St. George-Hyslop P. Homozygous and heterozygous *PINK1* mutations: considerations for diagnosis and care of Parkinson’s disease patients. Mov Disord. (2006) 21:875–9. doi: 10.1002/mds.20854, PMID: 16547921

[48] Norman BP Lubbe SJ Tan M Warren N Morris HR. Early onset Parkinson’s disease in a family of Moroccan origin caused by a p.A217D mutation in *PINK1*: a case report. BMC Neurol. (2017) 17:153. doi: 10.1186/s12883-017-0933-z 28789629 PMC5549325

[49] Biswas A Sadhukhan T Majumder S Misra AK Das SK Variation Consortium IG. Evaluation of *PINK1* variants in Indian Parkinson’s disease patients. Parkinsonism Relat Disord. (2010) 16:167–71. doi: 10.1016/j.parkreldis.2009.10.005 19889566

[50] Kilarski LL Pearson JP Newsway V Majounie E Knipe MDW Misbahuddin A. Systematic review and UK-based study of *PARK2 (parkin), PINK1, PARK7 (DJ-1)* and *LRRK2* in early-onset Parkinson’s disease: *PARK2, PINK1, PARK7, LRRK2* in EOPD. Mov Disord. (2012) 27:1522–9. doi: 10.1002/mds.25132, PMID: 22956510

[51] Tan EK Yew K Chua E Puvan K Shen H Lee E. *PINK1* mutations in sporadic early-onset Parkinson’s disease. Mov Disord. (2006) 21:789–93. doi: 10.1002/mds.20810 16482571

[52] Bouhouche A Tibar H Ben El Haj R El Bayad K Razine R Tazrout S. *LRRK2* G2019S mutation: prevalence and clinical features in Moroccans with Parkinson’s disease. Park Dis. (2017) 2017:1–7. doi: 10.1155/2017/2412486 PMC5390546 28465860

[53] Djarmati A Hedrich K Svetel M Lohnau T Schwinger E Romac S. Heterozygous*PINK1* mutations: a susceptibility factor for Parkinson disease? Mov Disord. (2006) 21:1526–30. doi: 10.1002/mds.20977 16755580

[54] Ibáñez P Lesage S Lohmann E Thobois S Michele GD Borg M. Mutational analysis of the *PINK1* gene in early-onset parkinsonism in Europe and North Africa. Brain. (2006) 129:686–94. doi: 10.1093/brain/awl005, PMID: 16401616

[55] Gierthmuhlen J Lienau F Maag R Hagenah JM Deuschl G Fritzer E. Somatosensory processing in a German family with *PINK1* mutations: its potential role in Parkinson disease. J Neurol Neurosurg Psychiatry. (2009) 80:571–4. doi: 10.1136/jnnp.2008.158659, PMID: 19372294

[56] Puschmann A Englund E Ross OA Vilariño-Güell C Lincoln SJ Kachergus JM. First neuropathological description of a patient with Parkinson’s disease and LRRK2 p.N1437H mutation. Parkinsonism Relat Disord. (2012) 18:332–8. doi: 10.1016/j.parkreldis.2011.11.019 22154298 PMC3330199

[57] Bras JM Guerreiro RJ Ribeiro MH Januario C Morgadinho A Oliveira CR. G2019S dardarin substitution is a common cause of Parkinson’s disease in a Portuguese cohort. Mov Disord. (2005) 20:1653–5. doi: 10.1002/mds.20682, PMID: 16149095

[58] Gosal D Ross OA Wiley J Irvine GB Johnston JA Toft M. Clinical traits of LRRK2-associated Parkinson’s disease in Ireland: a link between familial and idiopathic PD. Parkinsonism Relat Disord. (2005) 11:349–52. doi: 10.1016/j.parkreldis.2005.05.004, PMID: 16102999

[59] Hatano T Funayama M Kubo S Ichiro MIF Oji Y Mori A. Identification of a Japanese family with LRRK2 p.R1441G-related Parkinson’s disease. Neurobiol Aging. (2014) 35:2656.e17–23. doi: 10.1016/j.neurobiolaging.2014.05.025, PMID: 24973808 PMC4171438

[60] Khlebtovsky A Roditi Y Djaldetti R. Comparison of thermal sensation and pain thresholds in LRRK2 carriers and non carriers with Parkinson’s disease. (2023) Available at: https://www.mdsabstracts.org/abstract/comparison-of-thermal-sensation-and-pain-thresholds-in-lrrk2-carriers-and-non-carriers-with-parkinsons-disease/

[61] Hedrich K Winkler S Hagenah J Kabakci K Kasten M Schwinger E. RecurrentLRRK2 (Park8) mutations in early-onset Parkinson’s disease. Mov Disord. (2006) 21:1506–10. doi: 10.1002/mds.20990, PMID: 16758483

[62] Criscuolo C De Rosa A Guacci A Simons EJ Breedveld GJ Peluso S. The *LRRK2* R1441C mutation is more frequent than G2019S in Parkinson’s disease patients from southern Italy: *LRRK2* R1441C mutation more frequent in southern Italy. Mov Disord. (2011) 26:1732–6. doi: 10.1002/mds.23735, PMID: 21538529

[63] Li DW Gu Z Wang C Ma J Tang BS Chen SD. Non-motor symptoms in Chinese Parkinson’s disease patients with and without LRRK2 G2385R and R1628P variants. J Neural Transm. (2015) 122:661–7. doi: 10.1007/s00702-014-1281-4, PMID: 25062988

[64] Healy DG Falchi M O’Sullivan SS Bonifati V Durr A Bressman S. Phenotype, genotype, and worldwide genetic penetrance of LRRK2-associated Parkinson’s disease: a case-control study. Lancet Neurol. (2008) 7:583–90. doi: 10.1016/S1474-4422(08)70117-0, PMID: 18539534 PMC2832754

[65] Baig F Lawton M Rolinski M Ruffmann C Nithi K Evetts SG. Delineating nonmotor symptoms in early Parkinson’s disease and first-degree relatives. Mov Disord. (2015) 30:1759–66. doi: 10.1002/mds.26281, PMID: 26179331 PMC5034839

[66] An XK Peng R Li T Burgunder JM Wu Y Chen WJ. LRRK2 Gly2385Arg variant is a risk factor of Parkinson’s disease among Han-Chinese from mainland China. Eur J Neurol. (2008) 15:301–5. doi: 10.1111/j.1468-1331.2007.02052.x, PMID: 18201193

[67] Wang C Cai Y Gu Z Ma J Zheng Z Tang BS. Clinical profiles of Parkinson’s disease associated with common leucine-rich repeat kinase 2 and glucocerebrosidase genetic variants in Chinese individuals. Neurobiol Aging. (2014) 35:725.e1–6. doi: 10.1016/j.neurobiolaging.2013.08.012 24095219

[68] Zhang Z Burgunder JM An X Wu Y Chen W Zhang J. *LRRK2* R1628P variant is a risk factor of Parkinson’s disease among Han-Chinese from mainland China: LRRK2 R1628P variant. Mov Disord. (2009) 24:1902–5. doi: 10.1002/mds.22371 19672984

[69] Luciano MS Lipton RB Wang C Katz M Zimmerman ME Sanders AE. Clinical expression of *LRRK2* G2019S mutations in the elderly: clinical LRRK2 G2019S expression in the elderly. Mov Disord. (2010) 25:2571–6. doi: 10.1002/mds.23330, PMID: 20721910 PMC2978804

[70] Martino D Melzi V Franco G Kandasamy N Monfrini E Di Fonzo A. Juvenile dystonia-parkinsonism syndrome caused by a novel p.S941Tfs1X ATP13A2 (PARK9) mutation. Parkinsonism Relat Disord. (2015) 21:1378–80. doi: 10.1016/j.parkreldis.2015.09.036, PMID: 26421390

[71] Bonner N Bozzi S Morgan L Mason B Peterschmitt MJ Fischer TZ. Patients’ experiences of Parkinson’s disease: a qualitative study in glucocerebrosidase and idiopathic Parkinson’s disease. J Patient Rep Outcomes. (2020) 4:65. doi: 10.1186/s41687-020-00230-9, PMID: 32757092 PMC7406609

[72] Rodriguez-Porcel F Espay AJ Carecchio M. Parkinson disease in Gaucher disease. J Clin Mov Disord. (2017) 4:7. doi: 10.1186/s40734-017-0054-2, PMID: 28546865 PMC5440911

[73] Kresojević N Janković M Petrović I Kumar KR Dragašević N Dobričić V. Presenting symptoms of GBA-related Parkinson’s disease. Parkinsonism Relat Disord. (2015) 21:804–7. doi: 10.1016/j.parkreldis.2015.04.028, PMID: 25957717

[74] Jesús S Huertas I Bernal-Bernal I Bonilla-Toribio M Cáceres-Redondo MT Vargas-González L. GBA variants influence motor and non-motor features of Parkinson’s disease. PLoS One. (2016) 11:e0167749 doi: 10.1371/journal.pone.0167749 28030538 PMC5193380

[75] McNeill A Duran R Hughes DA Mehta A Schapira AHV. A clinical and family history study of Parkinson’s disease in heterozygous *glucocerebrosidase* mutation carriers. J Neurol Neurosurg Psychiatry. (2012) 83:853–4. doi: 10.1136/jnnp-2012-302402, PMID: 22577228 PMC3927562

[76] Neumann J Bras J Deas E O’Sullivan SS Parkkinen L Lachmann RH. Glucocerebrosidase mutations in clinical and pathologically proven Parkinson’s disease. Brain. (2009) 132:1783–94. doi: 10.1093/brain/awp044, PMID: 19286695 PMC2702833

[77] Deng H Wang P Jankovic J. The genetics of Parkinson disease. Ageing Res Rev. (2018) 42:72–85. doi: 10.1016/j.arr.2017.12.007 29288112

[78] Marras C Lang A van de Warrenburg BP Sue CM Tabrizi SJ Bertram L. Nomenclature of genetic movement disorders: recommendations of the international Parkinson and movement disorder society task force: nomenclature of genetic movement disorders. Mov Disord. (2016) 31:436–57. doi: 10.1002/mds.26527, PMID: 27079681

[79] Klein C Westenberger A. Genetics of Parkinson’s disease. Cold Spring Harb Perspect Med. (2012) 2:a008888–8. doi: 10.1101/cshperspect.a008888, PMID: 22315721 PMC3253033

[80] Li B Zhao G Zhou Q Xie Y Wang Z Fang Z. Gene4PD: a comprehensive genetic database of Parkinson’s disease. Front Neurosci. (2021) 15:679568. doi: 10.3389/fnins.2021.679568, PMID: 33981200 PMC8107430

[81] Elwan M Schaefer AM Craig K Hopton S Falkous G Blakely EL. Changing faces of mitochondrial disease: autosomal recessive *POLG* disease mimicking myasthenia gravis and progressive supranuclear palsy. BMJ Neurol Open. (2022) 4:e000352. doi: 10.1136/bmjno-2022-000352, PMID: 36518302 PMC9743281

[82] Bilguvar K Tyagi NK Ozkara C Tuysuz B Bakircioglu M Choi M. Recessive loss of function of the neuronal ubiquitin hydrolase UCHL1 leads to early-onset progressive neurodegeneration. Proc Natl Acad Sci. (2013) 110:3489–94. doi: 10.1073/pnas.1222732110, PMID: 23359680 PMC3587195

[83] Selvaraj S Piramanayagam S. Impact of gene mutation in the development of Parkinson’s disease. Genes Dis. (2019) 6:120–8. doi: 10.1016/j.gendis.2019.01.004, PMID: 31193965 PMC6545447

[84] Vivacqua G Yin JJ Casini A Li X Li YH D’Este L. Immunolocalization of alpha-synuclein in the rat spinal cord by two novel monoclonal antibodies. Neuroscience. (2009) 158:1478–87. doi: 10.1016/j.neuroscience.2008.12.001, PMID: 19118601

[85] Valek L Tran B Wilken-Schmitz A Trautmann S Heidler J Schmid T. Prodromal sensory neuropathy in *Pink1 ^−/−^ SNCA ^A53T^* double mutant Parkinson mice. Neuropathol Appl Neurobiol. (2021) 47:1060–79. doi: 10.1111/nan.12734, PMID: 33974284

[86] Johnson RA Kelm-Nelson CA Ciucci MR. Changes to ventilation, vocalization, and thermal nociception in the *Pink1*−/− rat model of Parkinson’s disease. J Parkinsons Dis. (2020) 10:489–504. doi: 10.3233/JPD-191853 32065805 PMC8142388

[87] Yi MH Shin J Shin N Yin Y Lee SY Kim CS. *PINK1* mediates spinal cord mitophagy in neuropathic pain. J Pain Res. (2019) 12:1685–99. doi: 10.2147/JPR.S198730, PMID: 31239755 PMC6554001

[88] Bichler Z Lim HC Zeng L Tan EK. Non-motor and motor features in LRRK2 transgenic mice. PLoS One. (2013) 8:e70249 doi: 10.1371/journal.pone.0070249 23936174 PMC3728021

[89] Valek L Auburger G Tegeder I. Sensory neuropathy and nociception in rodent models of Parkinson’s disease. Dis Model Mech. (2019) 12. doi: 10.1242/dmm.039396 PMC6602317 31248900

[90] Blauwendraat C Makarious MB Leonard HL Bandres-Ciga S Iwaki H Nalls MA. A population scale analysis of rare *SNCA* variation in the UK biobank. Neurobiol Dis. (2021) 148:105182. doi: 10.1016/j.nbd.2020.105182, PMID: 33307186 PMC7880248

[91] Giasson BI Duda JE Murray IVJ Chen Q Souza JM Hurtig HI. Oxidative damage linked to neurodegeneration by selective α-Synuclein nitration in Synucleinopathy lesions. Science. (2000) 290:985–9. doi: 10.1126/science.290.5493.985, PMID: 11062131

[92] Lotharius J. Impaired dopamine storage resulting from alpha-synuclein mutations may contribute to the pathogenesis of Parkinson’s disease. Hum Mol Genet. (2002) 11:2395–407. doi: 10.1093/hmg/11.20.2395, PMID: 12351575

[93] Lesage S Brice A. Parkinson’s disease: from monogenic forms to genetic susceptibility factors. Hum Mol Genet. (2009) 18:R48–59. doi: 10.1093/hmg/ddp012 19297401

[94] Hoffman-Zacharska D Koziorowski D Ross OA Milewski M Poznański J Jurek M. Novel A18T and pA29S substitutions in α-synuclein may be associated with sporadic Parkinson’s disease. Parkinsonism Relat Disord. (2013) 19:1057–60. doi: 10.1016/j.parkreldis.2013.07.011, PMID: 23916651 PMC4055791

[95] Kiely AP Asi YT Kara E Limousin P Ling H Lewis P. α-Synucleinopathy associated with G51D *SNCA* mutation: a link between Parkinson’s disease and multiple system atrophy? Acta Neuropathol (Berl). (2013) 125:753–69. doi: 10.1007/s00401-013-1096-7, PMID: 23404372 PMC3681325

[96] Krüger R Kuhn W Müller T Woitalla D Graeber M Kösel S. AlaSOPro mutation in the gene encoding α-synuclein in Parkinson’s disease. Nat Genet. (1998) 18:106–8. doi: 10.1038/ng0298-106 9462735

[97] Pasanen P Myllykangas L Siitonen M Raunio A Kaakkola S Lyytinen J. A novel α-synuclein mutation A53E associated with atypical multiple system atrophy and Parkinson’s disease-type pathology. Neurobiol Aging. (2014) 35:2180.e1–5. doi: 10.1016/j.neurobiolaging.2014.03.024, PMID: 24746362

[98] Polymeropoulos MH Lavedan C Leroy E Ide SE Dehejia A Dutra A. Mutation in the α-Synuclein gene identified in families with Parkinson’s disease. Science. (1997) 276:2045–7. doi: 10.1126/science.276.5321.2045 9197268

[99] Proukakis C Dudzik CG Brier T MacKay DS Cooper JM Millhauser GL. A novel -synuclein missense mutation in Parkinson disease. Neurology. (2013) 80:1062–4. doi: 10.1212/WNL.0b013e31828727ba, PMID: 23427326 PMC3653201

[100] Zarranz JJ Alegre J Gómez-Esteban JC Lezcano E Ros R Ampuero I. The new mutation, E46K, of α-synuclein causes parkinson and Lewy body dementia: new α-Synuclein gene mutation. Ann Neurol. (2004) 55:164–73. doi: 10.1002/ana.10795, PMID: 14755719

[101] Kasten M Klein C. The many faces of alpha-synuclein mutations: the many faces of alpha-Synuclein mutations. Mov Disord. (2013) 28:697–701. doi: 10.1002/mds.25499, PMID: 23674458

[102] Klein C Lohmann K. Parkinson disease(s): is “Parkin disease” a distinct clinical entity? Neurology. (2009) 72:106–7. doi: 10.1212/01.wnl.0000333666.65522.8d 18987349

[103] Klein C Schlossmacher MG. Parkinson disease, 10 years after its genetic revolution: multiple clues to a complex disorder. Neurology. (2007) 69:2093–104. doi: 10.1212/01.wnl.0000271880.27321.a7, PMID: 17761553

[104] Castelo Rueda MP Raftopoulou A Gögele M Borsche M Emmert D Fuchsberger C. Frequency of heterozygous Parkin (PRKN) variants and penetrance of Parkinson’s disease risk markers in the population-based CHRIS cohort. Front Neurol. (2021) 12:706145. doi: 10.3389/fneur.2021.706145, PMID: 34434164 PMC8382284

[105] Zhu W Huang X Yoon E Bandres-Ciga S Blauwendraat C Cade JH. Heterozygous *PRKN* mutations are common but do not increase the risk of Parkinson’s disease [internet]. Neurology. (2021). doi: 10.1101/2021.08.11.21261928 PMC9423714 35640906

[106] Kitada T Asakawa S Hattori N Matsumine H Yamamura Y Minoshima S. Mutations in the parkin gene cause autosomal recessive juvenile parkinsonism. Nature. (1998) 392:605–8. doi: 10.1038/33416, PMID: 9560156

[107] Klein C Schumacher K Jacobs H Hagenah J Kis B Garrels J. Association studies of Parkinson’s disease andparkin polymorphisms. Ann Neurol. (2000) 48:126–7. doi: 10.1002/1531-8249(200007)48:1<126::AID-ANA22>3.0.CO;2-K 10894229

[108] Hedrich K Eskelson C Wilmot B Marder K Harris J Garrels J. Distribution, type, and origin ofParkin mutations: review and case studies. Mov Disord. (2004) 19:1146–57. doi: 10.1002/mds.20234, PMID: 15390068

[109] Lesage S Lunati A Houot M Romdhan SB Clot F Tesson C. Characterization of recessive Parkinson disease in a large multicenter study. Ann Neurol. (2020) 88:843–50. doi: 10.1002/ana.25787 33045815 PMC8944279

[110] Dawson TM Dawson VL. The role of parkin in familial and sporadic Parkinson’s disease: the role of Parkin in familial and sporadic PD. Mov Disord. (2010) 25:S32–9. doi: 10.1002/mds.22798 20187240 PMC4115293

[111] Ishikawa A Tsuji S. Clinical analysis of 17 patients in 12 Japanese families with autosomal-recessive type juvenile parkinsonism. Neurology. (1996) 47:160–6. doi: 10.1212/WNL.47.1.160, PMID: 8710071

[112] Grünewald A Kasten M Ziegler A Klein C. Next-generation phenotyping using the *Parkin* example: time to catch up with genetics. JAMA Neurol. (2013) 70:1186. doi: 10.1001/jamaneurol.2013.488 23835509

[113] Valente EM Abou-Sleiman PM Caputo V Muqit MMK Harvey K Gispert S. Hereditary early-onset Parkinson’s disease caused by mutations in *PINK1*. Science. (2004) 304:1158–60. doi: 10.1126/science.1096284, PMID: 15087508

[114] Petit A Kawarai T Paitel E Sanjo N Maj M Scheid M. Wild-type *PINK1* prevents basal and induced neuronal apoptosis, a protective effect abrogated by Parkinson disease-related mutations. J Biol Chem. (2005) 280:34025–32. doi: 10.1074/jbc.M505143200, PMID: 16079129

[115] Bonifati V Rohe CF Breedveld GJ Fabrizio E De Mari M Tassorelli C. Early-onset parkinsonism associated with *PINK1* mutations: frequency, genotypes, and phenotypes. Neurology. (2005) 65:87–95. doi: 10.1212/01.wnl.0000167546.39375.82, PMID: 16009891

[116] Paisán Ruíz C Jain S Evans EW Gilks WP Simón J van der Brug M. Cloning of the gene containing mutations that cause PARK8-linked Parkinson’s disease. Neuron. (2004) 44:595–600. doi: 10.1016/j.neuron.2004.10.023 15541308

[117] Rui Q Ni H Li D Gao R Chen G. The role of LRRK2 in neurodegeneration of Parkinson disease. Curr Neuropharmacol. (2018) 16:1348–57. doi: 10.2174/1570159X16666180222165418 29473513 PMC6251048

[118] Nuytemans K Theuns J Cruts M Van Broeckhoven C. Genetic etiology of Parkinson disease associated with mutations in the *SNCA*, *PARK2*, *PINK1*, *PARK7*, and *LRRK2* genes: a mutation update. Hum Mutat. (2010) 31:763–80. doi: 10.1002/humu.21277, PMID: 20506312 PMC3056147

[119] Gehrke S Imai Y Sokol N Lu B. Pathogenic LRRK2 negatively regulates microRNA-mediated translational repression. Nature. (2010) 466:637–41. doi: 10.1038/nature09191, PMID: 20671708 PMC3049892

[120] Iwaki H Blauwendraat C Makarious MB Bandrés-Ciga S Leonard HL Gibbs JR. Penetrance of Parkinson’s disease in *LRRK2* p.G2019S carriers is modified by a polygenic risk score. Mov Disord. (2020 May) 35:774–80. doi: 10.1002/mds.27974, PMID: 31958187 PMC8975556

[121] Correia Guedes L Ferreira JJ Rosa MM Coelho M Bonifati V Sampaio C. Worldwide frequency of G2019S LRRK2 mutation in Parkinson’s disease: a systematic review. Parkinsonism Relat Disord. (2010) 16:237–42. doi: 10.1016/j.parkreldis.2009.11.004, PMID: 19945904

[122] Deng H Le W Guo Y Hunter CB Xie W Jankovic J. Genetic and clinical identification of Parkinson’s disease patients withLRRK2 G2019S mutation. Ann Neurol. (2005) 57:933–4. doi: 10.1002/ana.20510 15929036

[123] Alcalay RN Mejia-Santana H Mirelman A Saunders-Pullman R Raymond D Palmese C. Neuropsychological performance in LRRK2 G2019S carriers with Parkinson’s disease. Parkinsonism Relat Disord. (2015) 21:106–10. doi: 10.1016/j.parkreldis.2014.09.033, PMID: 25434972 PMC4306614

[124] Pankratz N Pauciulo MW Elsaesser VE Marek DK Halter CA Wojcieszek J. Mutations in DJ-1 are rare in familial Parkinson disease. Neurosci Lett. (2006) 408:209–13. doi: 10.1016/j.neulet.2006.09.003, PMID: 16997464 PMC1706076

[125] Bonifati V Rizzu P van Baren MJ Schaap O Breedveld GJ Krieger E. Mutations in the *DJ-1* gene associated with autosomal recessive early-onset parkinsonism. Science. (2003) 299:256–9. doi: 10.1126/science.1077209 12446870

[126] Meulener MC Xu K Thomson L Ischiropoulos H Bonini NM. Mutational analysis of DJ-1 in *Drosophila* implicates functional inactivation by oxidative damage and aging. Proc Natl Acad Sci. (2006) 103:12517–22. doi: 10.1073/pnas.0601891103, PMID: 16894167 PMC1533799

[127] Anderson PC Daggett V. Molecular basis for the structural instability of human DJ-1 induced by the L166P mutation associated with Parkinson’s disease. Biochemistry. (2008) 47:9380–93. doi: 10.1021/bi800677k, PMID: 18707128 PMC2646841

[128] Malgieri G Eliezer D. Structural effects of Parkinson’s disease linked DJ-1 mutations. Protein Sci. (2008) 17:855–68. doi: 10.1110/ps.073411608, PMID: 18436956 PMC2327288

[129] Weissbach A Wittke C Kasten M Klein C. ‘Atypical’ Parkinson’s disease – genetic. (2019) 207–235. Available at: https://linkinghub.elsevier.com/retrieve/pii/S0074774219300972 10.1016/bs.irn.2019.10.011 31779813

[130] Vilariño-Güell C Wider C Ross OA Dachsel JC Kachergus JM Lincoln SJ. VPS35 mutations in Parkinson disease. Am J Hum Genet. (2011) 89:162–7. doi: 10.1016/j.ajhg.2011.06.001, PMID: 21763482 PMC3135796

[131] Ando M Funayama M Li Y Kashihara K Murakami Y Ishizu N. *VPS35* mutation in Japanese patients with typical Parkinson’s disease: *VPS35* mutation in Japanese PD. Mov Disord. (2012) 27:1413–7. doi: 10.1002/mds.25145, PMID: 22991136

[132] Sharma M Ioannidis JPA Aasly JO Annesi G Brice A Bertram L. A multi-Centre clinico-genetic analysis of the *VPS35* gene in Parkinson disease indicates reduced penetrance for disease-associated variants. J Med Genet. (2012) 49:721–6. doi: 10.1136/jmedgenet-2012-101155, PMID: 23125461 PMC3488700

[133] Cerri S Ghezzi C Ongari G Croce S Avenali M Zangaglia R. GBA mutations influence the release and pathological effects of small extracellular vesicles from fibroblasts of patients with Parkinson’s disease. Int J Mol Sci. (2021) 22:2215. doi: 10.3390/ijms22042215, PMID: 33672321 PMC7927041

[134] Zhao F Bi L Wang W Wu X Li Y Gong F. Mutations of glucocerebrosidase gene and susceptibility to Parkinson’s disease: An updated meta-analysis in a European population. Neuroscience. (2016) 320:239–46. doi: 10.1016/j.neuroscience.2016.02.007, PMID: 26868973

[135] Beavan M McNeill A Proukakis C Hughes DA Mehta A Schapira AHV. Evolution of prodromal clinical markers of Parkinson disease in a *GBA* mutation–positive cohort. JAMA Neurol. (2015) 72:201–8. doi: 10.1001/jamaneurol.2014.2950, PMID: 25506732 PMC4326672

[136] Sidransky E Lopez G. The link between the GBA gene and parkinsonism. Lancet Neurol. (2012) 11:986–98. doi: 10.1016/S1474-4422(12)70190-4, PMID: 23079555 PMC4141416

[137] Granek Z Barczuk J Siwecka N Rozpędek-Kamińska W Kucharska E Majsterek I. GBA1 gene mutations in α-Synucleinopathies—molecular mechanisms underlying pathology and their clinical significance. Int J Mol Sci. (2023) 24:2044. doi: 10.3390/ijms24032044 36768367 PMC9917178

[138] Avenali M Blandini F Cerri S. Glucocerebrosidase defects as a major risk factor for Parkinson’s disease. Front Aging Neurosci. (2020) 12:97. doi: 10.3389/fnagi.2020.00097, PMID: 32372943 PMC7186450

[139] Velez-Pardo C Lorenzo-Betancor O Jimenez-Del-Rio M Moreno S Lopera F Cornejo-Olivas M. The distribution and risk effect of GBA variants in a large cohort of PD patients from Colombia and Peru. Parkinsonism Relat Disord. (2019) 63:204–8. doi: 10.1016/j.parkreldis.2019.01.030, PMID: 30765263 PMC7175776

[140] Ryan E Seehra G Sharma P Sidransky E. GBA1-associated parkinsonism: new insights and therapeutic opportunities. Curr Opin Neurol. (2019) 32:589–96. doi: 10.1097/WCO.0000000000000715, PMID: 31188151

[141] Riboldi GM Di Fonzo AB. GBA, Gaucher disease, and Parkinson’s disease: from genetic to clinic to new therapeutic approaches. Cells. (2019) 8:364. doi: 10.3390/cells8040364 31010158 PMC6523296

[142] Tayebi N. Gaucher disease with parkinsonian manifestations: does glucocerebrosidase deficiency contribute to a vulnerability to parkinsonism? Mol Genet Metab. (2003) 79:104–9. doi: 10.1016/S1096-7192(03)00071-4, PMID: 12809640

[143] Lill CM. Genetics of Parkinson’s disease. Mol Cell Probes. (2016) 30:386–96. doi: 10.1016/j.mcp.2016.11.001 27818248

[144] Ramirez A Heimbach A Gründemann J Stiller B Hampshire D Cid LP. Hereditary parkinsonism with dementia is caused by mutations in ATP13A2, encoding a lysosomal type 5 P-type ATPase. Nat Genet. (2006) 38:1184–91. doi: 10.1038/ng1884, PMID: 16964263

[145] Tan J Zhang T Jiang L Chi J Hu D Pan Q. Regulation of intracellular manganese homeostasis by Kufor-Rakeb syndrome-associated ATP13A2 protein. J Biol Chem. (2011) 286:29654–62. doi: 10.1074/jbc.M111.233874, PMID: 21724849 PMC3191006

[146] Park JS Blair NF Sue CM. The role of ATP13A2 in Parkinson’s disease: clinical phenotypes and molecular mechanisms: ATP13A2 in Parkinson’s disease. Mov Disord. (2015) 30:770–9. doi: 10.1002/mds.26243 25900096

[147] Vidyadhara DJ Somayaji M Wade N Yücel B Zhao H Shashaank N. Dopamine transporter and synaptic vesicle sorting defects underlie auxilin-associated Parkinson’s disease. Cell Rep. (2023) 42:112231. doi: 10.1016/j.celrep.2023.112231, PMID: 36920906 PMC10127800

[148] Yim YI Sun T Wu LG Raimondi A De Camilli P Eisenberg E. Endocytosis and clathrin-uncoating defects at synapses of auxilin knockout mice. Proc Natl Acad Sci. (2010) 107:4412–7. doi: 10.1073/pnas.1000738107, PMID: 20160091 PMC2840126

[149] Edvardson S Cinnamon Y Ta-Shma A Shaag A Yim YI Zenvirt S. A deleterious mutation in *DNAJC6* encoding the neuronal-specific Clathrin-Uncoating co-chaperone Auxilin, is associated with juvenile parkinsonism. PLoS One. (2012) 7:e36458 doi: 10.1371/journal.pone.0036458 22563501 PMC3341348

[150] Köroğlu Ç Baysal L Cetinkaya M Karasoy H Tolun A. *DNAJC6* is responsible for juvenile parkinsonism with phenotypic variability. Parkinsonism Relat Disord. (2013) 19:320–4. doi: 10.1016/j.parkreldis.2012.11.006, PMID: 23211418

[151] Olgiati S Quadri M Fang M Rood JPMA Saute JA Chien HF. *D NAJC 6* mutations associated with early-onset Parkinson’s disease: *DNAJC6* mutations in Parkinson’s disease. Ann Neurol. (2016) 79:244–56. doi: 10.1002/ana.24553, PMID: 26528954

[152] Elsayed LEO Drouet V Usenko T Mohammed IN Hamed AAA Elseed MA. A novel nonsense mutation in *DNAJC 6* expands the phenotype of autosomal-recessive juvenile-onset Parkinson’s disease. Ann Neurol. (2016) 79:335–7. doi: 10.1002/ana.24591, PMID: 26703368

[153] Ng J Cortès-Saladelafont E Abela L Termsarasab P Mankad K Sudhakar S. *DNAJC6* mutations disrupt dopamine homeostasis in juvenile Parkinsonism-Dystonia. Mov Disord. (2020) 35:1357–68. doi: 10.1002/mds.28063, PMID: 32472658 PMC8425408

[154] Kipreos ET Pagano M. The F-box protein family. Genome Biol. (2000) 1:REVIEWS3002. doi: 10.1186/gb-2000-1-5-reviews3002, PMID: 11178263 PMC138887

[155] Wei L Ding L Li H Lin Y Dai Y Xu X. Juvenile-onset parkinsonism with pyramidal signs due to compound heterozygous mutations in the F-box only protein 7 gene. Parkinsonism Relat Disord. (2018) 47:76–9. doi: 10.1016/j.parkreldis.2017.11.332, PMID: 29174172

[156] Shojaee S Sina F Banihosseini SS Kazemi MH Kalhor R Shahidi GA. Genome-wide linkage analysis of a parkinsonian-pyramidal syndrome pedigree by 500 K SNP arrays. Am J Hum Genet. (2008) 82:1375–84. doi: 10.1016/j.ajhg.2008.05.005, PMID: 18513678 PMC2427312

[157] Fonzo AD Dekker MCJ Montagna P Baruzzi A Yonova EH Guedes LC. FBXO7 mutations cause autosomal recessive, early-onset parkinsonian-pyramidal syndrome. Neurology. (2009) 72:240–5. doi: 10.1212/01.wnl.0000338144.10967.2b, PMID: 19038853

[158] Paisán-Ruiz C Guevara R Federoff M Hanagasi H Sina F Elahi E. Early-onset L-dopa-responsive parkinsonism with pyramidal signs due to *ATP13A2, PLA2G6, FBXO7* and *spatacsin* mutations: complex recessive Parkinsonisms. Mov Disord. (2010) 25:1791–800. doi: 10.1002/mds.23221, PMID: 20669327 PMC6005705

[159] Keller Sarmiento IJ Afshari M Kinsley L Silani V Akhtar RS Simuni T. Novel bi-allelic FBXO7 variants in a family with early-onset typical Parkinson’s disease. Parkinsonism Relat Disord. (2022) 104:88–90. doi: 10.1016/j.parkreldis.2022.10.014, PMID: 36274328

[160] Gündüz A Eken AG Bilgiç B Hanagasi HA Bilgüvar K Günel M. FBXO7–R498X mutation: phenotypic variability from chorea to early onset parkinsonism within a family. Parkinsonism Relat Disord. (2014) 20:1253–6. doi: 10.1016/j.parkreldis.2014.07.016 25169713

[161] Conedera S Apaydin H Li Y Yoshino H Ikeda A Matsushima T. FBXO7 mutations in Parkinson’s disease and multiple system atrophy. Neurobiol Aging. (2016) 40:192.e1–5. doi: 10.1016/j.neurobiolaging.2016.01.003, PMID: 26882974

[162] Lohmann E Coquel AS Honoré A Gurvit H Hanagasi H Emre M. A new F-box protein 7 GENE MUTATION CAUSING TYPICAL Parkinson’s disease: *FBXO7* GENE MUTATION CAUSING TYPICAL PD. Mov Disord. (2015) 30:1130–3. doi: 10.1002/mds.26266, PMID: 26010069

[163] Lesage S Mangone G Tesson C Bertrand H Benmahdjoub M Kesraoui S. Clinical variability of SYNJ1-associated early-onset parkinsonism. Front Neurol. (2021) 12:648457. doi: 10.3389/fneur.2021.648457, PMID: 33841314 PMC8027075

[164] Quadri M Fang M Picillo M Olgiati S Breedveld GJ Graafland J. Mutation in the *SYNJ1* gene associated with autosomal recessive. Early-Onset Parkinsonism Hum Mutat. (2013) 34:1208–15. doi: 10.1002/humu.22373 23804577

[165] Krebs CE Karkheiran S Powell JC Cao M Makarov V Darvish H. The Sac1 domain of *SYNJ 1* identified mutated in a family with early-onset progressive P arkinsonism with generalized seizures. Hum Mutat. (2013) 34:1200–7. doi: 10.1002/humu.22372, PMID: 23804563 PMC3790461

[166] Klatt-Schreiner K Valek L Kang J Khlebtovsky A Trautmann S Hahnefeld L. High glucosylceramides and low anandamide contribute to sensory loss and pain in Parkinson’s disease. Mov Disord. (2020) 35:1822–33. doi: 10.1002/mds.28186, PMID: 32652698

[167] Alcalay RN Levy OA Waters CH Fahn S Ford B Kuo SH. Glucocerebrosidase activity in Parkinson’s disease with and without *GBA* mutations. Brain. (2015) 138:2648–58. doi: 10.1093/brain/awv179, PMID: 26117366 PMC4564023

[168] Buhidma Y Rukavina K Chaudhuri KR Duty S. Potential of animal models for advancing the understanding and treatment of pain in Parkinson’s disease. Npj Park Dis. (2020) 6:1. doi: 10.1038/s41531-019-0104-6, PMID: 31934609 PMC6944694

[169] George AJ Hoffiz YC Charles AJ Zhu Y Mabb AM. A comprehensive atlas of E3 ubiquitin ligase mutations in neurological disorders. Front Genet. (2018) 9:29. doi: 10.3389/fgene.2018.00029, PMID: 29491882 PMC5817383

